# Natural variation in *Arabidopsis thaliana* Cd responses and the detection of quantitative trait loci affecting Cd tolerance

**DOI:** 10.1038/s41598-017-03540-z

**Published:** 2017-06-16

**Authors:** Sina Fischer, Thomas Spielau, Stephan Clemens

**Affiliations:** 1 Department of Plant Physiology, Bayreuth Center of Ecology and Environmental Research, University of Bayreuth, Universitätsstrasse 30, 95447 Bayreuth, Germany; 2 School of Biosciences, University of Nottingham, Sutton Bonington Campus, Loughborough, Leicestershire LE12 5RD UK

## Abstract

Metal tolerance is often a result of metal storage or distribution. Thus, with the goal of advancing the molecular understanding of such metal homeostatic mechanisms, natural variation of metal tolerance in *Arabidopsis thaliana* was investigated. Substantial variation exists in tolerance of excess copper (Cu), zinc (Zn) and cadmium (Cd). Two accessions, Col-0 and Bur-0, and a recombinant inbred line (RIL) population derived from these parents were chosen for further analysis of Cd and Zn tolerance variation, which is evident at different plant ages in various experimental systems and appears to be genetically linked. Three QTLs, explaining in total nearly 50% of the variation in Cd tolerance, were mapped. The one obvious candidate gene in the mapped intervals, *HMA3*, is unlikely to contribute to the variation. In order to identify additional candidate genes the Cd responses of Col-0 and Bur-0 were compared at the transcriptome level. The sustained common Cd response of the two accessions was dominated by processes implicated in plant pathogen defense. Accession-specific differences suggested a more efficient activation of acclimative responses as underlying the higher Cd tolerance of Bur-0. The second hypothesis derived from the physiological characterization of the accessions is a reduced Cd accumulation in Bur-0.

## Introduction

Plant traits show enormous intraspecific natural variation. Molecular dissection of this variation can lead to new mechanistic insights into physiological or developmental processes and ideally yield an understanding of local adaptation to specific habitats. Most traits vary quantitatively and are controlled by multiple genes^1^. *Arabidopsis thaliana* represents an ideal system for molecular studies of natural variation because (i) it is distributed across a wide range of environments, and (ii) tremendous molecular knowledge and a vast array of genetic tools are available^2^. Accordingly, the natural variation in many different traits has already been studied in *A. thaliana*, enabling major progress in the understanding of diverse biological phenomena such as control of flowering time or herbivore defense^3^.

An important aspect of a plant’s environment is the composition of the soil. Plant roots are exposed to a huge range of as well as rapid fluctuations in the concentrations of bioavailable mineral ions. Relevant as environmental factors are not only macro- and microelements such as phosphorus or iron (Fe), but also potentially highly toxic elements without biological function, for example cadmium (Cd). Nonessential toxic elements are present in the environment either because of natural causes or because of man-made pollution. Cd has been released into the environment by industrial activities (e.g. metal smelting, battery manufacturing) and agricultural practices (e.g. use of phosphate fertilizers and sewage sludge)^4, 5^.

Plants have to acquire essential microelements (Fe, Zn, Cu, Mn, Co, Ni, Mo, B) from soil solutions that can vary in concentrations of the respective ions by orders of magnitude. Not only deficiency is a threat but also toxicity. An excess of, for instance, Cu or Zn ions can inhibit growth due to the tendency of these metal ions to strongly interact with various cellular components^6^. A homeostatic system comprising metal transporters, metal ligands and regulatory proteins maintains the concentrations of essential elements within rather narrow physiological ranges inside plant tissues and limits the accumulation of non-physiological elements^7, 8^. Variation in the so-called ionome, the mineral element composition of a tissue, an organ or an organism^9^, is the result of complex physiological processes and interactions with the environment^10^. Consequently, molecular dissection of ionome variation has led to a better functional understanding of metal transporters^11^, for example the Mo transporter MOT1^12^ or the metal efflux protein FPN2/IREG2^13^.

Natural variation in metal tolerance, i.e. the ability to withstand an excess of essential or nonessential metal ions, has received less attention, probably because metal excess is comparatively rare under natural conditions. However, many genes contributing to metal tolerance are part of the homeostatic network controlling metal accumulation and distribution. The Zn tolerance gene *ZIF1* encodes a nicotianamine transporter and affects translocation of Zn and Fe^14^. The major plant metal tolerance mechanism, metal-activated synthesis of phytochelatins, has an influence on Zn mobility within plants^15^. Thus, analysis of metal tolerance variation can potentially improve the understanding of metal homeostasis and this has already been demonstrated. One of the earliest studies on variation in *A. thaliana* metal tolerance revealed a nonfunctional allele of the P-type ATPase gene *HMA5* as the underlying cause of Cu hypersensitivity in the accession Cvi-0^16^. The exploration of variation in Zn tolerance^17^ improved the functional understanding of FRD3, a transporter mediating the loading of metal chelating citrate into the xylem^18^. Variation in the recently identified arsenate (AsV) reductase ATQ1/HAC1 controls both differences in arsenate tolerance^19^ and the shoot accumulation of As^20^.

In search of novel plant metal tolerance/metal homeostasis factors we therefore explored natural variation in Zn, Cu and Cd tolerance. Our data confirmed the existence of substantial differences between *A. thaliana* accessions in metal tolerance and motivated a genetic analysis of Cd tolerance variation. We identified QTLs contributing to variation in the growth response to Cd exposure in a Col-0 × Bur-0 recombinant inbred line population. In addition we compared the transcriptome changes upon Cd exposure in these two parental accessions in order to find candidate genes for the major QTL on chromosome 5 and to probe the extent of variation in Cd responses between *A. thaliana* accessions.

## Results

### Detection of natural variation in *A. thaliana* metal tolerance

**Figure 1:**
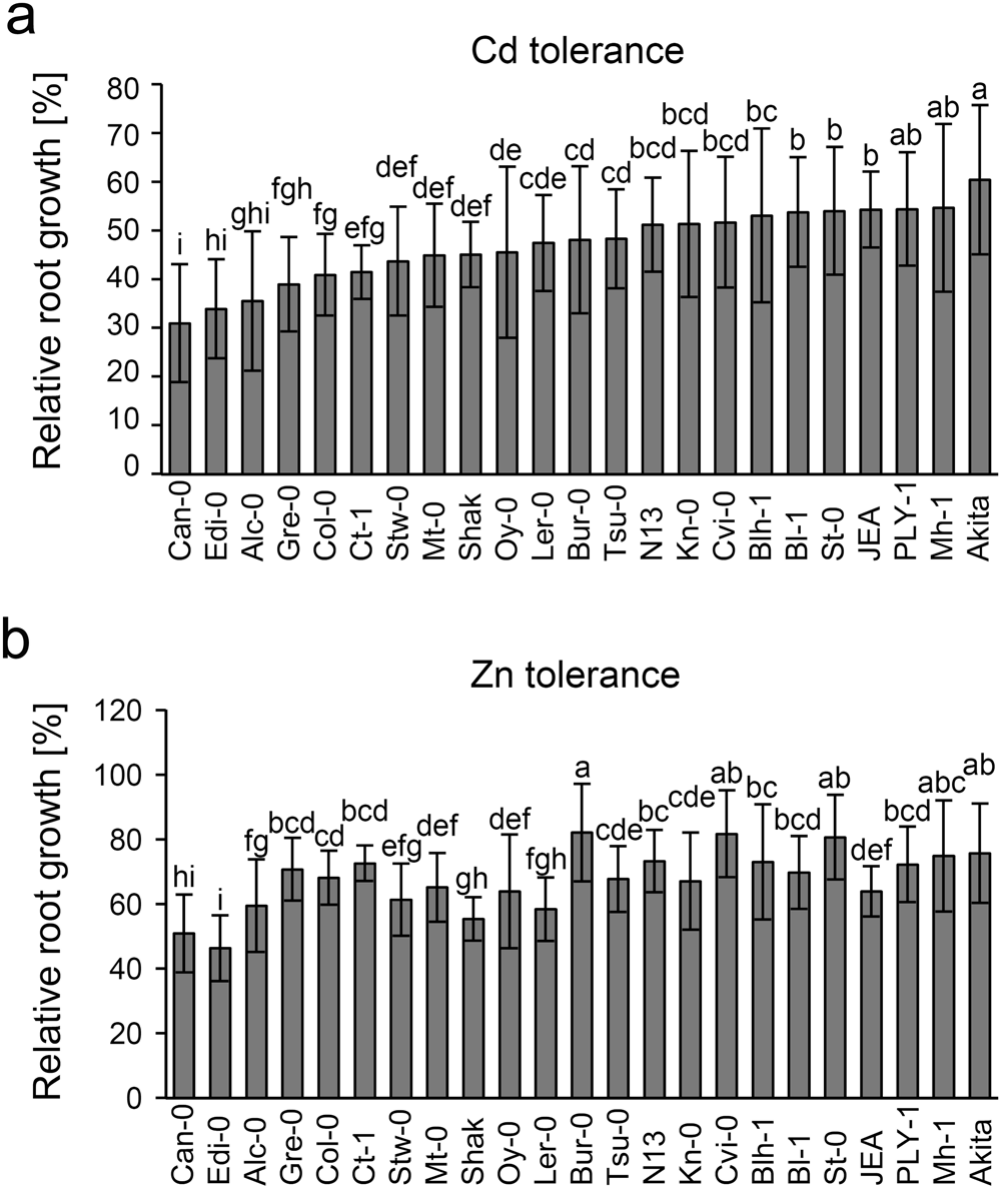
Natural variation in *A. thaliana* Zn and Cd tolerance. 23 accessions of the Versailles Core24 were grown on vertical agar plates under control conditions or in the presence of either 5 µM CdCl_2_ (**a**) or 50 µM ZnSO_4_ (**b**). Length of primary roots was determined after 12–14 d. Relative root growth of seedlings as an indicator for tolerance was calculated as follows: root length metal treatment/mean root length control conditions × 100. Absolute root lengths (in mm) under control conditions were: Bl-1: 64 ± 17; Stw-0:66 ± 26; N13: 69 ± 26; Akita: 70 ± 18; Shak: 77 ± 19; St-0: 82 ± 14; Blh-1: 83 ± 16; Ct-1: 84 ± 19; Kn-0: 85 ± 20; Can-0: 85 ± 16; Mh-1: 87 ± 18; Col-0: 88 ± 15; Ply-1: 90 ± 22; Cvi-0: 90 ± 14; Oy-0: 91 ± 23; Edi-0: 91 ± 16; JEA: 92 ± 22; Tsu-0: 94 ± 21; Ler-0: 94 ± 24; Mt-0: 96 ± 17; Bur-0: 99 ± 22; Gre-0: 102 ± 23; Alc-0: 103 ± 20). Data represent means ± SD. Per accession 17 to 185 plants were compared. The average n was 119. Statistical analysis was performed via one-way ANOVA and data were grouped based on Tukey’s 95% confidence intervals.

We tested a panel of *A. thaliana* accessions, assembled to maximize genetic diversity (23 out of the Core 24^21^), for their tolerance against an excess of the micronutrients Zn and Cu as well as growth-inhibiting concentrations of Cd. Relative root growth (RRG) of metal-treated plants was used as readout to assess tolerance. We detected variation in Cd tolerance ranging from 31 ± 5 to 60 ± 12% RRG in the presence of Cd. Zn tolerance varied to a similar degree with extremes of 46 ± 17 and 82 ± 18% relative growth (Fig. 1). Huge variation was detected in Cu tolerance because of the extreme Cu-sensitivity of Cvi-0 (7.5 ± 3.4%) (Supplementary Fig. S1) which is attributable to a loss of HMA5 function^16^.

**Figure 2:**
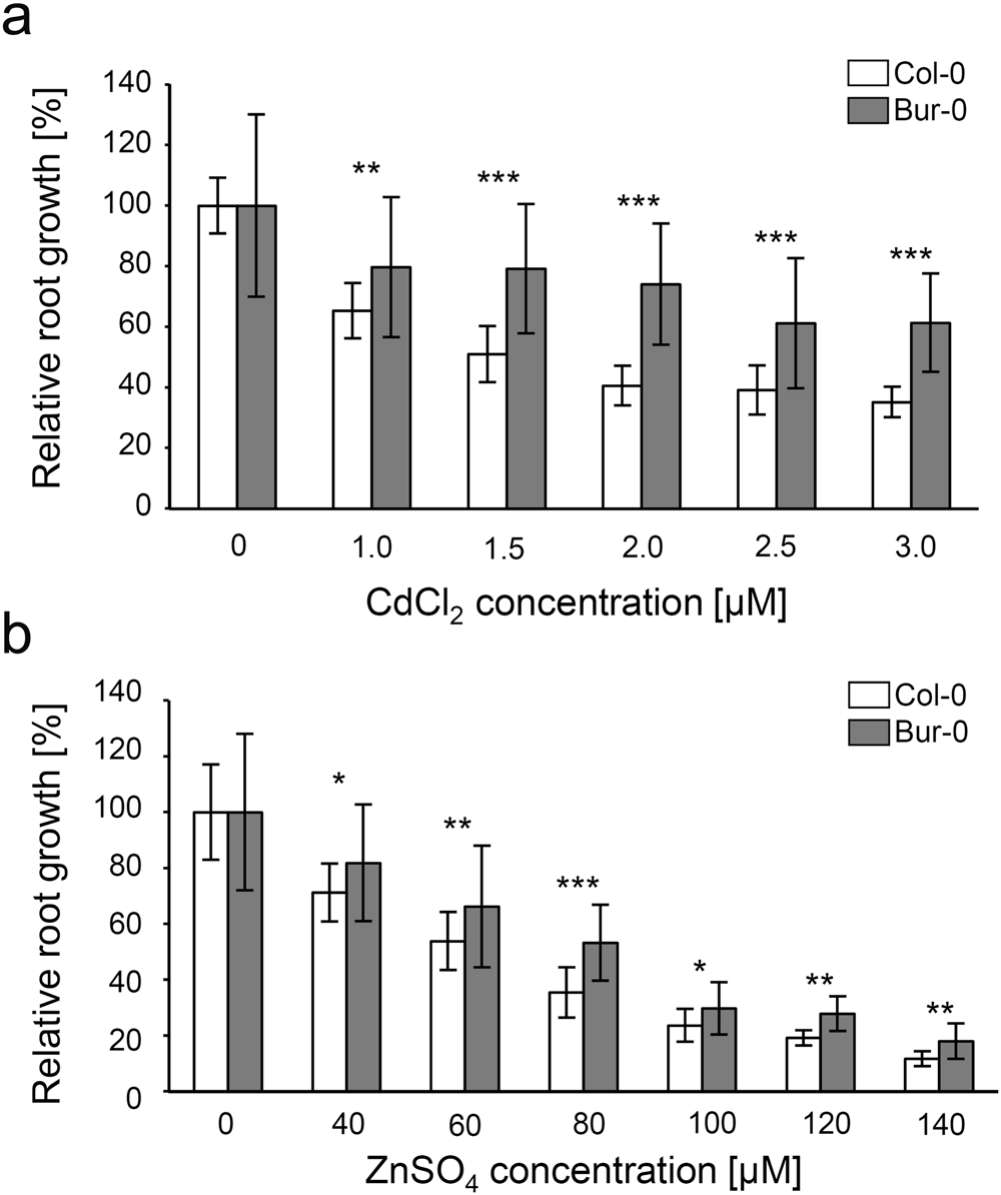
Accessions Col-0 and Bur-0 differ in their root growth response to toxic concentrations of Zn^2+^ and Cd^2+^. Col-0 (white bars) and Bur-0 (grey bars) seedlings were grown on vertical agar plates under control conditions (0 µM extra metal added) or in the presence of either varying concentrations of CdCl_2_ (**a**) or ZnSO_4_ (**b**) Relative root growth of seedlings as an indicator for tolerance was calculated as follows: root length metal treatment/mean root length control conditions × 100. In (**a**) absolute root lengths (in mm) under control conditions were: for Col-0 84 ± 7.6, for Bur-0 80 ± 24.1. Data represent means ± SD of 40–70 individuals grown in two independent experiments. In (**b**) absolute root lengths (in mm) under control conditions were: Col-0 85 ± 15, Bur-0 96 ± 27. Data represent means ± SD of 18–74 individuals grown in 2–4 independent experiments. Significant differences between accessions are indicated by asterisks (***P < 0.001; **P < 0.01; *P < 0.05) and were calculated by one-way ANOVA and subsequent Tukey test, 95% confidence interval.

With the aim of dissecting the genetic basis for the observed natural variation in *A. thaliana* metal tolerance, we focused on a comparison of the accessions Col-0 and Bur-0. This choice was governed by the difference in Zn and Cd tolerance with Bur-0 showing better relative growth in the presence of both metals and, more importantly, the availability of a recombinant inbred line (RIL) population at the time^22^. First, Zn and Cd tolerance of the two parental accessions were assessed more closely. Both showed similar root growth in control conditions and a dose-dependent growth reduction when treated with Cd or excess Zn (Fig. 2). At 1.5 µM CdCl_2_ the difference in the RRG of Col-0 and Bur-0 became highly significant and largest differences were observed upon addition of 2 µM CdCl_2_ to the medium. Bur-0 reached 74 ± 20% of the length under control conditions (80 ± 24.1 mm) while Col-0 reached only 41 ± 7% of 84 ± 7.6 mm. Under excess Zn conditions the accessions showed significant differences at concentrations ranging from 40 to 140 µM, with highly significant differences in RRG at 80 µM. Here Bur-0 displayed a growth of 53 ± 14%. Col-0 reached only 35 ± 9% RRG.

**Figure 3:**
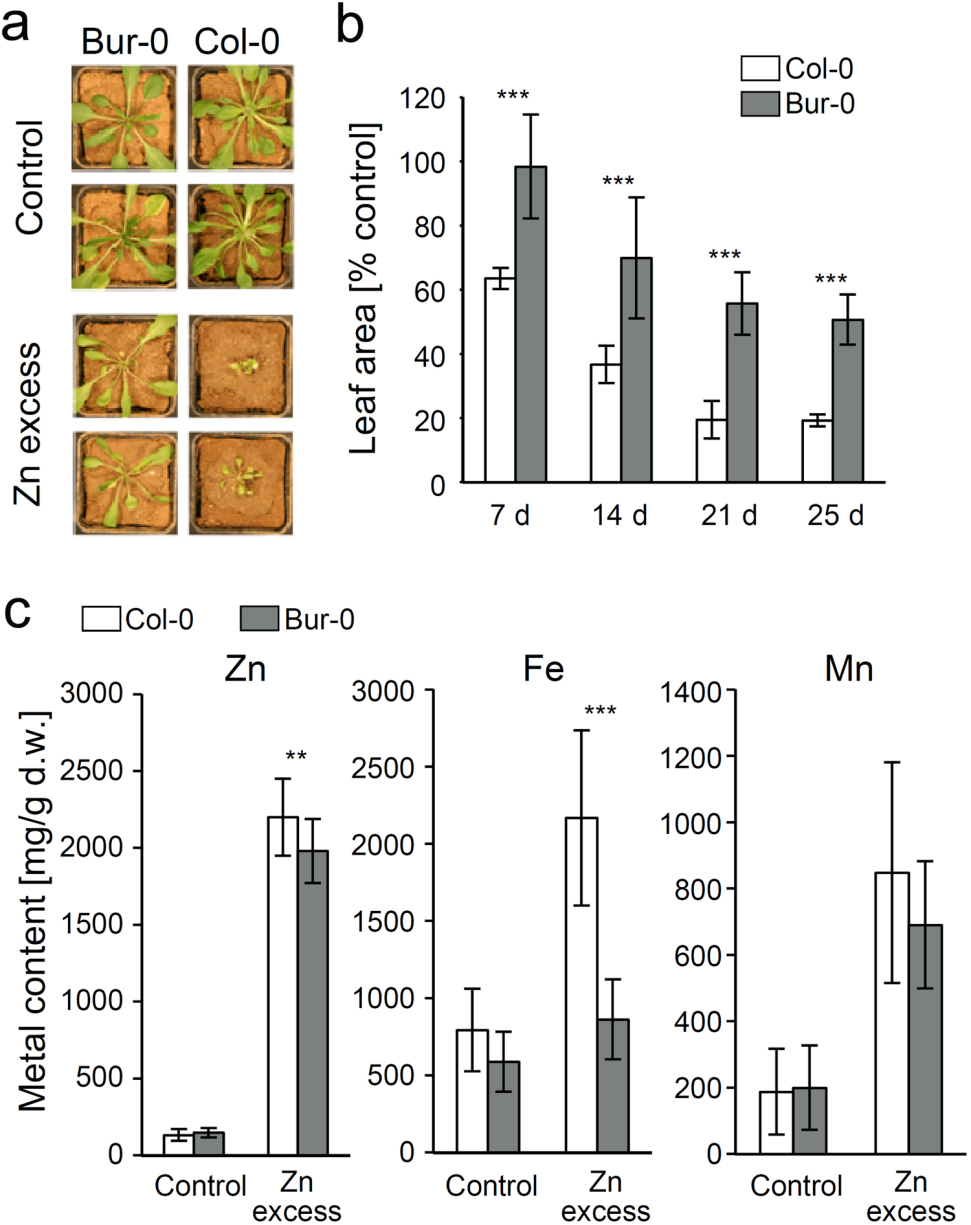
Growth and metal accumulation of accessions Bur-0 and Col-0 are differentially affected by an excess of Zn in the soil. Seven day old Col-0 and Bur-0 seedlings were transferred into soil with contrasting Zn supply. Pictures in (**a**) show plants after 25 d of growth in either control soil (400 mg ZnSO_4_ added per kg soil, 52 µg/g dry soil HCl-extractable Zn) or Zn excess soil (6400 mg ZnSO_4_ added per kg soil, 756 µg/g dry soil HCl-extractable Zn). (**b**): Growth of Col-0 (white bars) and Bur-0 (grey bars) was tracked by quantifying the leaf area of single plants over the course of the experiment. Relative growth for each individual on Zn excess soil was calculated as follows: Leaf area/mean leaf area control soil × 100. Data represent means ± SD of 3 independent experiments with each >10 individual plants per accession and growth condition. (**c**): Metal content of leaves after 25 d of growth on the two contrasting soils. Data represent means ± SD of 3 independent experiments (n = 9, i.e. three sample pools per plant line and condition). Statistical analysis was performed by one-way ANOVA and subsequent Tukey test. The asterisks indicate significant differences between the accessions (***P < 0.001; **P < 0.01), 95% confidence interval. Percentage values were square root transformed prior to statistical analysis.

Variation in metal tolerance observed for seedlings grown *in vitro* does not necessarily apply also to more natural metal excess conditions in soil. Therefore, Col-0 and Bur-0 were exemplarily analyzed in control and in Zn excess soil. Extractable metal content of the soil was analyzed via ICP-OES after HCl extraction. With Fe contents of 168 and 233 µg/g dry soil, Mn contents of 142 and 155 µg/g dry soil as well as Al contents of 267 and 294 µg/g dry soil, similar levels of metal ions could be detected in control and Zn-spiked soil. Zn levels were 52 and 756 µg/g dry soil in control and Zn-spiked soil, respectively. Growth was monitored over the course of the experiment by measuring leaf area (Fig. 3). In control soil the two accessions did not differ significantly. In Zn excess soil, however, Bur-0 showed much better growth than Col-0. After 7 d the relative leaf area (RLA) (% of control) was still at 98 ± 16% for Bur-0 while for Col-0 it had already dropped to 64 ± 4%. At the end of the experiment after 25 d the RLA of Bur-0 was reduced to 50 ± 7% while Col-0 plants showed an RLA of only 19 ± 1%, significantly less than Bur-0. Zn accumulation in leaves of plants grown in Zn excess soil was slightly lower in Bur-0 while Fe accumulation was strongly reduced compared to Col-0. Mn accumulation did not differ between the two accessions (Fig. 3c).

**Figure 4:**
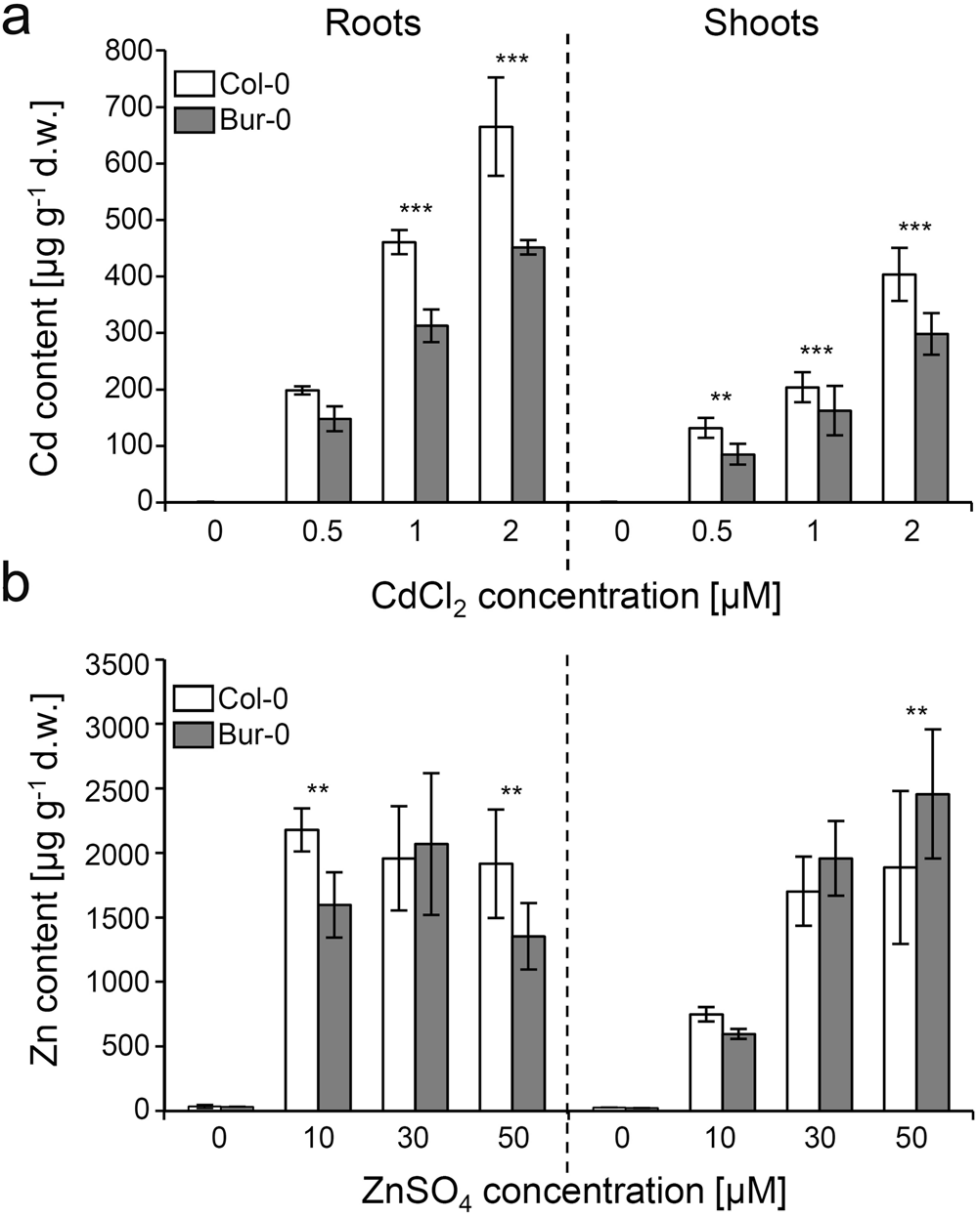
Cd and Zn accumulation differ in hydroponically cultivated Col-0 and Bur-0 plants. Cd (**a**) and Zn (**b**) content of hydroponically grown Col-0 (white bars) and Bur-0 (grey bars) plants was determined in roots (left) and shoots (right) after six weeks of growth. For the last week of cultivation, different concentrations of CdCl_2_ and ZnSO_4_, respectively, were added to the medium. Data represent means ± SD of 3 independent experiments (n = 6, i.e. two sample pools per plant line and condition). Statistical analysis was performed by one-way ANOVA and subsequent Tukey test. The asterisks indicate significant differences between the accessions (***P < 0.001; **P < 0.01), 95% confidence interval.

Final step in the phenotypic analysis of the contrasting accessions was the analysis of metal accumulation in hydroponically grown plants, i.e. under conditions that provide easy access to root tissue. For plants grown in medium with varying Cd concentrations lower Cd accumulation was consistently observed for Bur-0 relative to Col-0 in both roots and shoots (Fig. 4a). Zn differences between the accessions were less pronounced with Bur-0 showing slightly lower Zn levels in roots and higher levels in shoots at the highest tested Zn exposure (Fig. 4b). The micronutrients Fe and Mn were also assessed (Supplementary Fig. S2). Fe contents of Bur-0 roots were lower under all tested conditions. No other consistent differences in metal content between the accessions were found.

### Genetic analysis of the difference in Cd tolerance between Col-0 and Bur-0

**Figure 5:**
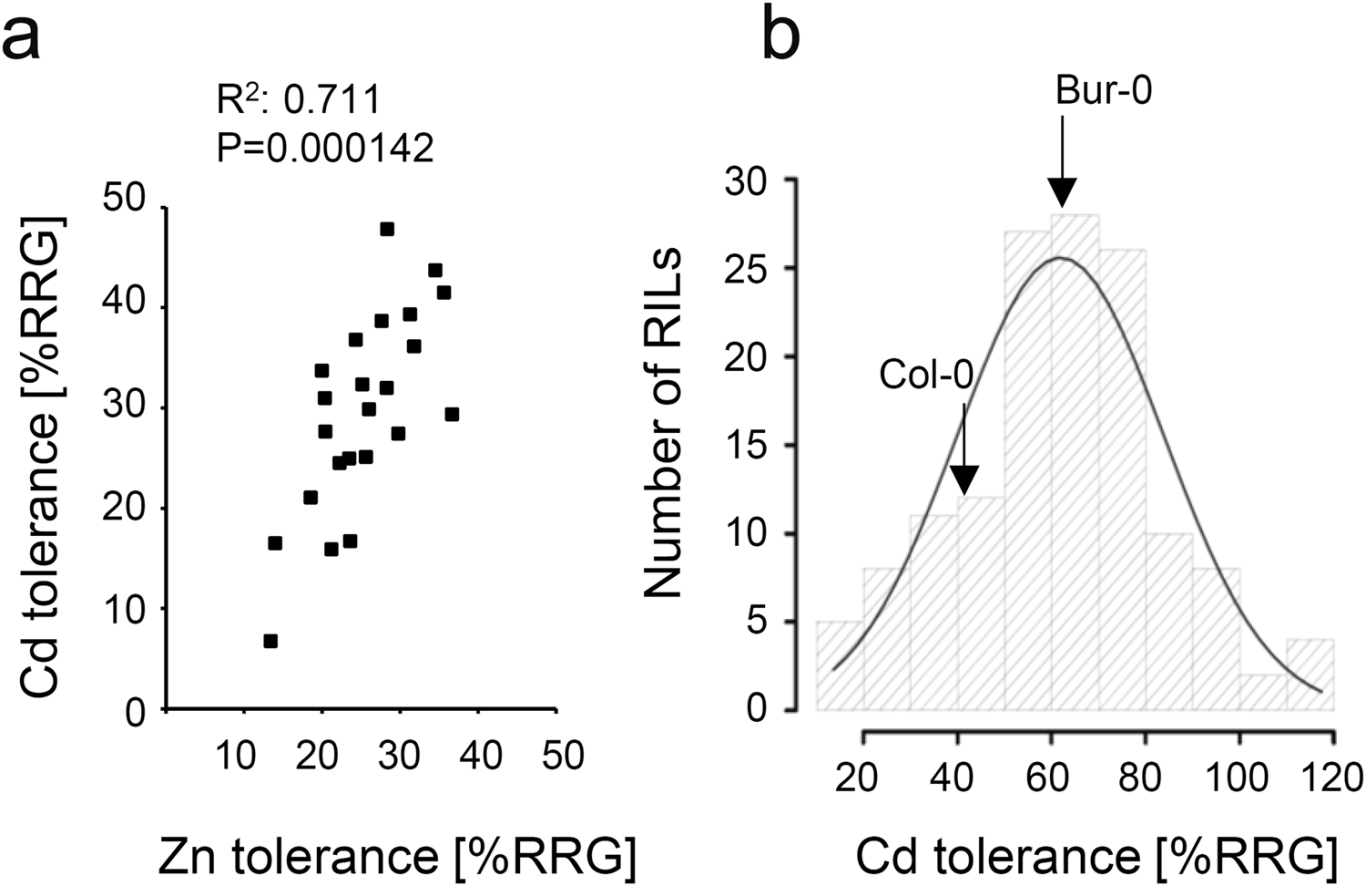
Natural variation of Cd tolerance and its correlation with Zn tolerance in a Bur-0 × Col-0 RIL population. (**a**) Pearson product moment correlation of Zn and Cd tolerance, assessed as % relative root growth (%RRG), of 21 randomly chosen Bur-0 × Col-0 RILs and the two parent accessions. (**b**) Frequency distribution and fitted normal distribution of Cd tolerance among the tested 131 Bur-0 × Col-0 RILs.

In order to assess whether the differences in Cd and Zn tolerance of seedlings are possibly linked, we initially analyzed 21 randomly selected RILs of the Bur-0 × Col-0 RIL population. The mean RRG under Zn and Cd stress conditions was strongly correlated (r = 0.7, *P* < 0.001) (Fig. 5a) indicating that differences in Cd and Zn tolerance between Col-0 and Bur-0 might be attributable to variation in the same genes. For the mapping of QTLs underlying natural variation in metal tolerance we then focused on Cd tolerance because the respective phenotypic difference between Col-0 and Bur-0 was more pronounced and also more robust than the difference in Zn tolerance (see Fig. 2).

Growth of the RIL population was assessed at 2 µM CdCl_2_ stress since the growth difference between Bur-0 and Col-0 was most prominent at this concentration (Fig. 2b). In 14 separate assays conducted to test the Cd tolerance of all RILs, Bur-0 and Col-0 showed a mean RRG of 65 ± 10% and 43 ± 10%, respectively. Of the 164 RILs 131 could be included in the mapping analysis. The germination frequency of the other 33 lines was too low to ensure accurate determination of the Cd tolerance. The RRG of the 131 selected lines was normally distributed and varied from 13.5 to 117.5% (Fig. 5b).

**Figure 6:**
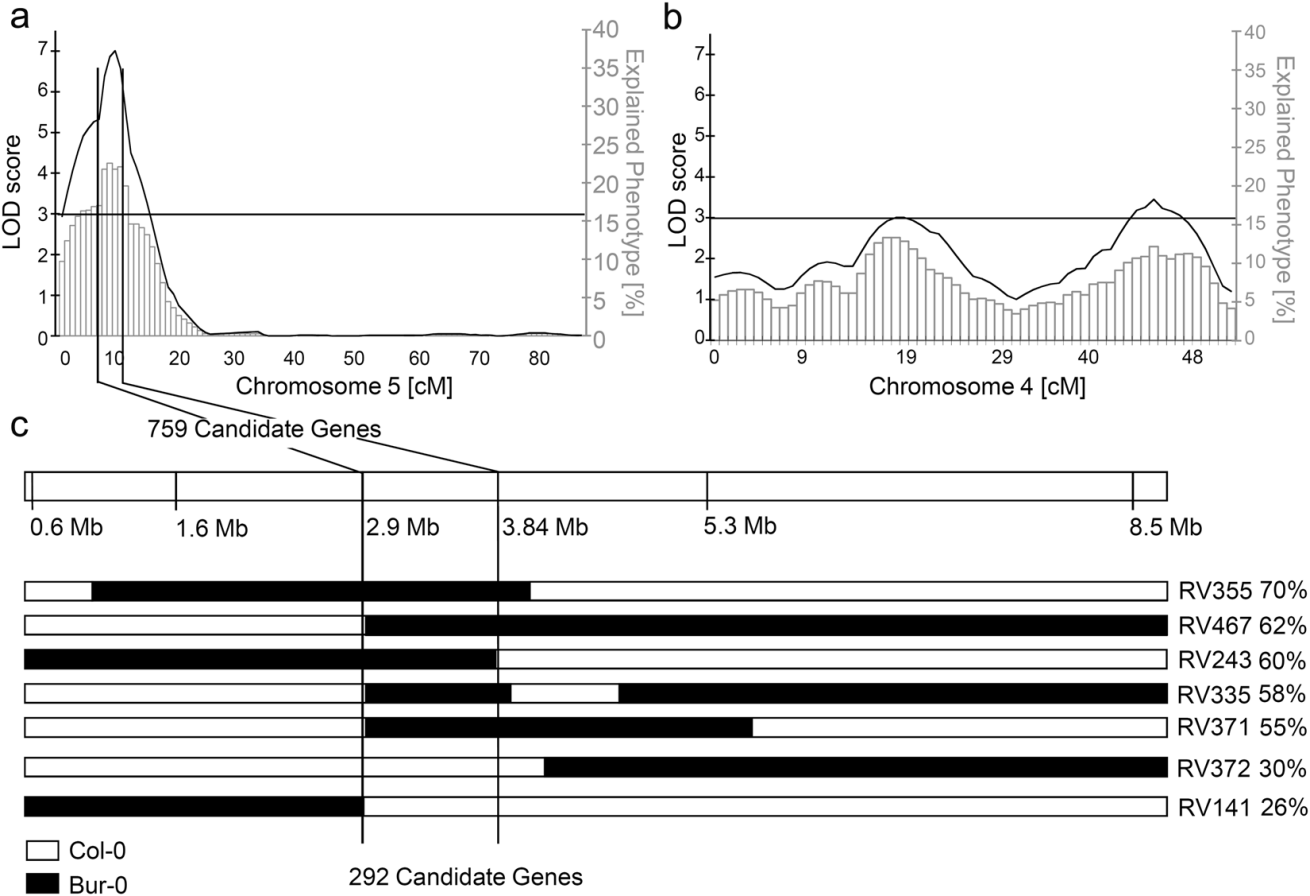
Genetic linkage mapping of loci contributing to variation in Cd tolerance across the Bur-0 × Col-0 RIL population. QTL mapping with MapQTL5 after determination of the relative root growth of 131 RILs at 2 µM CdCl_2_ detected a major QTL on chromosome 5 (**a**) and two minor QTLs on chromosome 4 (**b**). (**c**) Through additional marker analysis the QTL on chromosome 5 was narrowed to an interval located between 2.9 and 4.01 Mb. Indicated are the genotypes (white: Col-0; black Bur-0) and the Cd tolerance (in %RRG) for informative RILs.

The program MapQTL5^®^ was used to correlate genotypic and phenotypic information of all RILs. QTLs with a logarithm of odds (LOD) score of greater than 3 (95% confidence interval as calculated by MapQTL5^®^) were detected on chromosome 4 between 5.6 and 6.9 Mb as well as 13.2 and 17.7 Mb, and on chromosome 5 between 2.9 and 5.3 Mb (Fig. 6a,b). These QTLs explained 16, 12 and 23%, respectively, of the difference in Cd tolerance between Col-0 and Bur-0. Using the relative seedling weight as tolerance readout, QTL regions were detected in the same positions, albeit with less statistical significance (Supplementary Fig. S3). The mapped QTL regions contain 355, 299 and 759 genes. The only gene located within the QTLs that was previously implicated in Cd tolerance is *HMA3* (At4G30120). This heavy metal ATPase was shown to influence natural variation in Cd accumulation in *A. thaliana* shoots^23^, and HMA3 overexpression resulted in a slight increase in Cd tolerance^24^. Col-0 is known to carry a defective *HMA3* allele^25^. We therefore transformed Col-0 with the *HMA3*_Bur-0_ allele. However, no consistent increase in Cd tolerance was observed when independent transgenic lines were compared to Col-0. The minor increase in Cd tolerance in one transgenic line did not correlate with the expression level of *HMA3*_*Bur-0*_ (Supplementary Fig. S4), indicating that a functional HMA3 does not alleviate Cd toxicity under our assay conditions. Instead, other genes must be responsible for the observed difference in Cd tolerance between Bur-0 and Col-0. Even the phytochelatin-deficient Cd hypersensitive *cad1-3* mutant in Col-0 background showed only a minor, non-significant gain in Cd tolerance when expressing the *HMA3*_Bur-0_ allele (Supplementary Fig. S4).

Further analysis was focused on the QTL on chromosome 5 since it had the largest effect on the phenotypic difference between Bur-0 and Col-0. RILs containing mainly Bur-0 alleles in this QTL region were selected. Genetic markers were generated for the interval between 2.9 and 5.3 Mb and then used to narrow the QTL interval to between 2.9 and 3.84 Mb (Fig. 6c). Within this region, between At5g09370 and At5G11900, 292 genes are located. A comparison with a list of 243 *A. thaliana* genes annotated as metal homeostasis-related^26^ showed that none of these is positioned in this interval.

### Identification of putative candidate genes

Because of this lack of obvious candidate genes we employed two different strategies to find the genes most plausibly involved in Cd tolerance. First, we searched for genes in the mapped interval that contain polymorphisms between the two accessions^27, 28^. SNPs and indels in coding regions were highlighted and those resulting in an amino acid exchange were prioritized. In 155 genes a total of 395 such SNPs were detected. They were analyzed for their putative impact on protein functionality. A SIFT analysis^29, 30^ revealed that 51 genes carry nonsynonymous SNPs or indels predicted to be deleterious for protein function (Supplementary Table S1). The Bur-0 genome apparently carries polymorphisms possibly rendering 49 of these proteins nonfunctional. Two proteins were predicted to be functional in Bur-0 but not in Col-0, including the Mg transporter MGT7 (At5g09690). One gene appears to be nonfunctional in both accessions (At5g09730).

In our initial screen we observed that Cvi-0 and St-0 showed levels of Zn and Cd tolerance similar to Bur-0 (Fig. 1). While QTLs are of course population-dependent we nonetheless analyzed all genes of the QTL region for their functionality in Cvi-0 and St-0 and compared the results to the SIFT analysis for Bur-0. This comparison showed that of the two genes which are predicted to be non-functional in Col-0 but functional in Bur-0, only MGT7 is also predicted to be functional in Cvi-0 and St-0 highlighting it as a candidate for the observed QTL. Furthermore 11 of the 49 genes, predicted to be non-functional in Bur-0, are also predicted as non-functional in Cvi-0 and St-0 (Supplementary Table S2).

The second strategy searched for expression differences between Col-0 and Bur-0 in genes present in the mapped interval. A microarray experiment was conducted to find Cd responsive genes and to search for differential expression in Bur-0 and Col-0. Because this analysis is genome-wide it additionally provided information on natural variation of transcriptional responses to Cd exposure in *A. thaliana*. A comparatively mild Cd treatment was chosen in order to avoid confounding effects through differences in the severity of stress for the two accessions. Hydroponic growth in medium with 1 µM CdCl_2_ resulted in comparable growth reduction as determined by RLA: 57 ± 11% for Bur-0 and 58 ± 5% for Col-0 (Supplementary Fig. S5). As a sensitive biochemical marker for Cd exposure we analyzed the accumulation of PCs. PC synthesis is activated by cytosolic Cd and therefore can be used as readout for biologically active symplastic Cd levels. Comparable amounts of PC2 and PC3 accumulated in roots and shoots of Col-0 and Bur-0 during the Cd exposure indicating equal activation of AtPCS1 (Supplementary Fig. S5). Transcriptome changes in roots as the tissue of immediate Cd impact were analyzed using the ATH1 chip. For 183 genes in the QTL interval expression was detected. Out of these 35 genes showed differences between Col-0 and Bur-0. They either responded only in Bur-0 or only in Col-0, or were responsive in both accessions but showed at least a twofold difference in transcript level between the accessions under control or treatment conditions (Supplementary Fig. S6). For example, At5g09570 encoding a Cox19-like protein of unknown function showed higher expression level under control conditions and was more Cd responsive in Bur-0. Transcript abundance of the putative oligopeptide transporter gene At5g11570 was Cd-repressed only in Col-0.

### Natural variation in *A. thaliana* Cd responses

**Figure 7:**
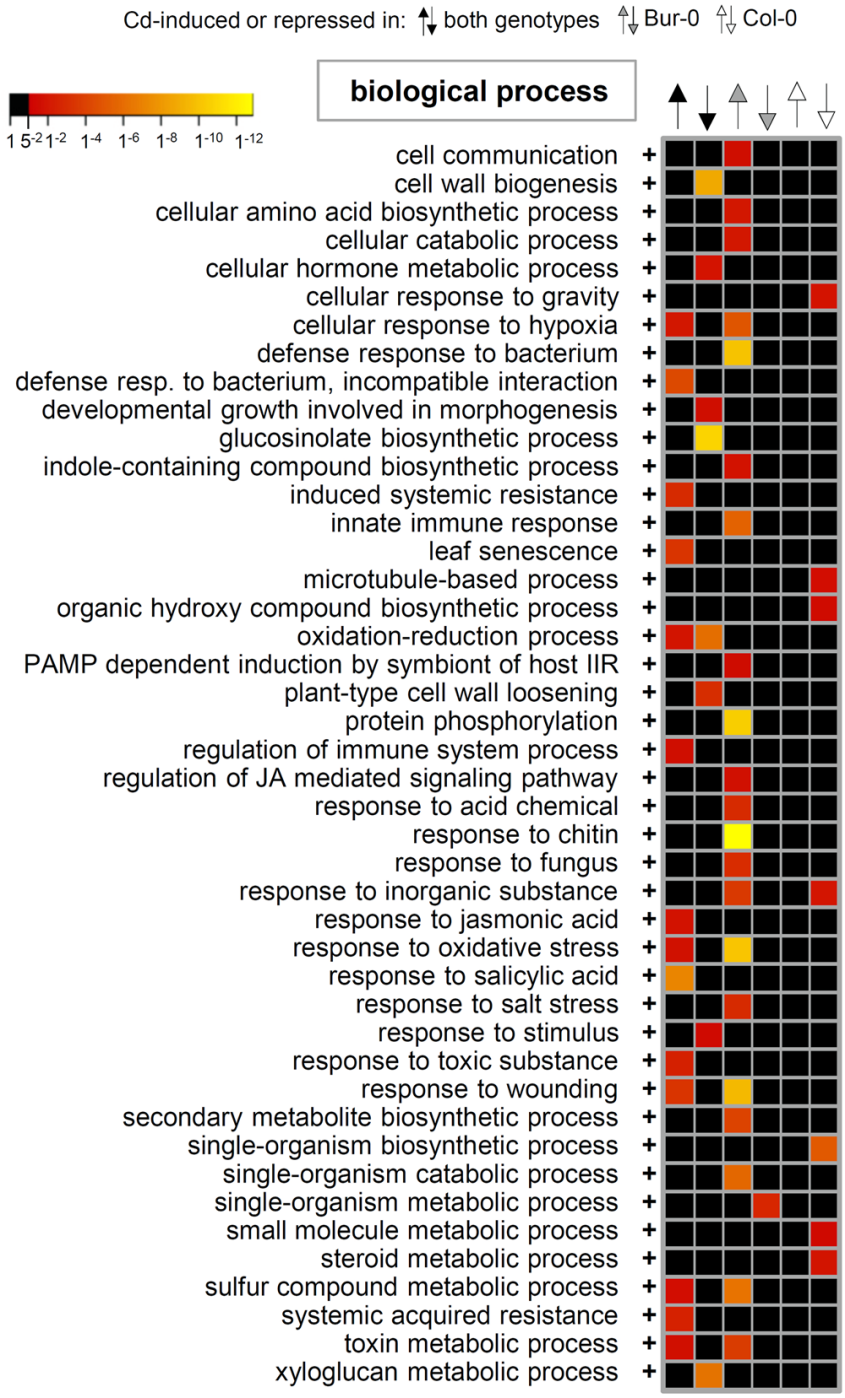
GO terms overrepresented in common or accession-specific Cd responses. Genes with a ≥2fold response (*P*_adj_ < 0.05) to Cd treatment in both accessions (black arrows for induction or repression under Cd exposure) or in only one accession (Col-0: white arrows; Bur-0: grey arrows) were selected for GO term enrichment analysis. Depicted are *P*_adj_. values (Bonferroni correction) for the most defined biological processes over-represented in at least one category; no under-representations were found (complete results of the Panther analysis can be found in Supplementary dataset S2).

The transcriptome data enabled global analysis of variation in Cd response. In total the expression of 16,022 genes was analyzed in root tissue. Fold changes were determined for each gene after Cd treatment and expression was compared between Bur-0 and Col-0. A differential expression was defined as a ≥2fold change of expression and a *P*_adj_ of <0.05. In Col-0 698 genes were up-regulated upon Cd treatment and 736 were down-regulated. In Bur-0 more genes were Cd responsive, namely 1090 were up- and 722 were down-regulated. 522 and 388 genes, respectively, responded qualitatively in the same way in both accessions (Supplementary dataset S1). An enrichment analysis of the functional classes of those 2336 genes was performed and significant over- or underrepresentation determined (Fig. 7 and Supplementary dataset S2). In this way an *A. thaliana* Cd core response can be defined. Several biological processes activated in both accessions (left column in Fig. 7) are related to immune responses, e.g. “systemic acquired resistance”, “induced systemic resistance”, “response to jasmonic acid”, “response to salicylic acid”, “defense response to bacterium, incompatible interaction”. Part of the Cd core response was also the up-regulation of abiotic stress-associated processes such as “cellular response to hypoxia”, “response to oxidative stress” and “response to toxic substance”. Furthermore, several genes belonging to the GO category “sulfur compound metabolic process” were more active in both Col-0 and Bur-0 upon Cd exposure. A common theme of GO terms enriched among down-regulated genes was the cell wall: “cell wall biogenesis”, plant-type cell wall loosening”, and “xyloglucan metabolic process”. Also down-regulated was “glucosinolate biosynthetic process”. Several GO terms were specifically enriched among the genes responsive only in Bur-0. Most of them represent additional biological processes involved in pathogen defense, e.g. “innate immune response”, “response to chitin”, “response to fungus”. In contrast, no GO terms were enriched in the group of genes specifically up-regulated in Col-0.

## Discussion

Cd as an environmental pollutant is almost ubiquitously present at the global scale due to diffuse pollution over centuries. Important sources include metal smelting and the application of phosphate fertilizers with Cd impurities^4^. Humans are chronically exposed to low levels of Cd mostly through the consumption of plant-derived food because plants tend to take up and accumulate Cd^5^. Recent risk assessments, for instance by the European Food Safety Authority (EFSA), concluded that (i) the provisional tolerable weekly intake of Cd should be lowered to reduce health risks such as renal damage, osteoporosis, cardiovascular disease, and that (ii) current levels of human exposure to Cd are on average around this suggested lowered threshold of 2.5 µg/kg body weight (b.w.). Therefore, environmental exposure to Cd needs to be reduced (EFSA 2009, 2012). An efficient way of achieving this would be the development of crops with strongly reduced Cd accumulation rates, which in turn would benefit from a mechanistic understanding of genes and proteins mediating the movement of Cd within a plant. Because accumulation rates of metals and the ability to cope with an excess of the same metals are often tightly linked, e.g. in the case of the arsenate reductase ATQ1/HAC1^19, 20^, the elucidation of tolerance mechanisms represents one way of identifying genes involved in the handling of Cd by plants.

Forward genetic screens were instrumental in elucidating the molecular basis of plant Cd tolerance mechanisms. The analysis of *A. thaliana cad* mutants demonstrated the key role of the phytochelatin pathway for Cd detoxification. Mutants with defects in glutathione or phytochelatin biosynthesis are strongly Cd hypersensitive^31^. We aimed at finding new factors contributing to Cd tolerance by studying natural variation within *A. thaliana*.

While some early reports on metal tolerance variation could not be confirmed^32^ we found within a core set of genetically diverse *A. thaliana* accessions^21^ about twofold variation for Zn and Cd tolerance at the seedling stage. In the case of Zn this value is between the variation factors of 1.6 and 4.4 reported for the highest and the lowest growth inhibitory Zn concentrations, respectively, applied to a very similar set of *A. thaliana* accessions^17^. More in-depth analyses of two accessions, Col-0 and Bur-0, indicated that differences in Zn tolerance are far stronger when plants are cultivated in Zn excess soil (Fig. 3). Bur-0 showed much better growth than Col-0. Corresponding experiments with Cd were not performed for safety reasons as the addition of growth-inhibitory Cd concentrations to soil could result in the exposure of experimenters to Cd-containing dust. Because Cd and Zn tolerance variation appear to be linked (Fig. 5a), the analysis of Cd tolerance variation may well reveal hidden factors of Zn homeostasis as was discussed for HMA3. The existence of functional and nonfunctional *HMA3* alleles explains variation in leaf Cd accumulation. There is also a contribution to Zn accumulation differences but this is much weaker, most likely because Zn accumulation is influenced by more genes than Cd accumulation^23^.

A possible explanation of higher Bur-0 Cd tolerance is reduced Cd accumulation relative to Col-0. In hydroponically grown plants root and shoot Cd contents were consistently lower in Bur-0 (Fig. 4), again illustrating the connection between tolerance and accumulation. For Zn the situation is less clear. In Zn excess soil Bur-0 leaves contained slightly less Zn than Col-0. On the other hand, the situation in hydroponic culture is less clear with trends depending on tissue and metal concentration. In leaves Bur-0 accumulated more Zn while in roots a lower Zn accumulation was visible at the highest concentration the plants were exposed to. Data from the IonomicsHub (Baxter 2007) suggest that leaves of soil-grown Bur-0 plants accumulate less Cd than Col-0, which would be consistent with findings reported here. Publicly available data for two trays (868, 1060) with Col-0 and Bur-0 showed 30% and 9% less Cd for Bur-0 (ionomicshub. org). Zn content varied much less between the accessions (0 to 12% in trays 868, 1060, 1301, and 1482), again consistent with our observations.

The most extreme phenotypic difference found between Col-0 and Bur-0 in our study, namely the dramatically better leaf growth of Bur-0 on Zn excess soil, may at least in part be due to the higher Fe accumulation of Col-0 under these conditions and thus Fe toxicity (Fig. 3c). However, the mechanistic basis for this difference in Fe accumulation is unclear.

Analysis of Cd tolerance across the Bur-0 × Col-0 RIL population revealed strong variation for this trait (Fig. 5b). Normal distribution indicated the contribution of several genetic factors to the variation as was observed for two other RIL populations, Ler-0 × Col-4 and Col-gl1 × Kas-1, before^33^. Three QTLs could be mapped, the major one on chromosome 5 explaining 23% of the variation. Not surprisingly, the mapped intervals do not overlap with the previously mapped QTLs regions contributing to variation in the above-mentioned RIL populations^33^.

A large number of QTLs involved in abiotic stress tolerance have been detected in *A. thaliana* to date. However, rather few have been molecularly elucidated^34^. As was the case here, the mapped intervals are usually several Mb wide. Molecular elucidation of QTLs is therefore largely dependent on whether or not genes that have previously been implicated in the trait in question are located in the mapped interval. Examples of successful identification of the causal polymorphisms include *APR2* for sulfate content^35^, *MOT1* for Mo accumulation^12^, and *FRD3* for Zn tolerance^18^. Within the three mapped Cd tolerance QTLs only HMA3 had been implicated in Cd accumulation^23^ and tolerance^24^ before. The latter was not confirmed by the data presented here. Transformation of Col-0 or even the Cd hypersensitive mutant *cad1-3*, which both lack a functional *HMA3* allele due to a deletion of one nucleotide in exon 8, with the Bur-0 allele of *HMA3* under control of its own promotor did not enhance Cd tolerance. This discrepancy is possibly attributable to expression differences as the previously reported effect of *HMA3* on Cd tolerance was conferred by constitutive overexpression. Overall, the proposed contribution of *HMA3* polymorphisms to variation in *A. thaliana* Cd tolerance^24^ appears to be absent across the Bur-0 × Col-0 RIL population.

Further fine-mapping of the major QTL on chromosome 5 using an F2 population derived from a cross of RIL 243 with Col-0 failed because we did not find a sufficient number of recombination events in the Bur-0 region spanning the QTL. Thus, in the absence of other obvious candidate genes we attempted to find potential candidate genes (i) by evaluating polymorphisms in the mapped region between At5g09350 and At5g11950, and (ii) by comparing the transcriptional responses of Col-0 and Bur-0 to Cd exposure. The former strategy was based on the analysis of nonsynonymous SNPs and indels. It suggested the Mg transporter genes AT5G09690 (*MGT7*/*MRS2-7*) and AT5G09720 for further study given the known effect of Mg status on Cd tolerance^36^. The status of *MGT7* as a candidate gene is further supported by the SIFT analyses for the accessions Cvi-0 and St-0 (Supplementary Table S2) which showed Bur-0-like growth responses to Cd and Zn toxicity.

The analysis of global transcriptome changes in the roots of Col-0 and Bur-0 was carried out with older, hydroponically grown plants. This ensured access to sufficient root material and allowed comparative assessment of the degree of Cd stress on the two accessions. Accumulation of PCs is a suitable indicator for intracellular, biologically active Cd because PC synthesis, which is mostly attributable to AtPCS1, is strongly activated by the presence of Cd. Levels of the two major phytochelatins, PC2 and PC3, were similar for roots and shoots of Col-0 and Bur-0 (Supplementary Fig. S5). This demonstrated comparable stress levels. Also, in order to avoid variation caused by slight variation in kinetics of the Cd responses we analyzed a comparatively late time point, i.e. after one week of exposure. Within the QTL region on chromosome 5 several genes were found to be responsive to Cd. These included the sulfate transporter *SULTR2;1* gene (At5g10180) and At5g10380 (encoding a putative E3 ligase) which were previously found to be robustly Cd-responsive in roots at earlier time points (2, 6, and 30 h)^37^. However, based on current knowledge, no obvious new candidate genes emerged from the transcript analysis of genes in the chromosome 5 interval.

A genome-wide comparison of the Col-0 and Bur-0 root Cd responses can help define transcriptome changes conserved in *A. thaliana*. The focus of our analysis was on sustained acclimative changes. This is complementary to available data for much earlier time points between 2 h and 30 h (e.g. refs 37–39). Nonetheless substantial overlap between the core response shown by Col-0 and Bur-0 and more rapid transcriptome changes is apparent. Among the 540 up-regulated and 400 down-regulated genes constituting the Cd core response reported here, 36 and 39%, respectively, showed corresponding changes in roots of 4 week old hydroponically grown *A. thaliana* Col-0 plants exposed to Cd^2+^ for 30 h^37^. Similarly, 38% of the genes found to be up-regulated in roots of 10 d old *A. thaliana* Col-4 seedlings exposed for 24 to Cd^2+^ ^40^ are on the list of common Cd-induced genes in Col-0 and Bur-0. Thus, a considerable fraction of the acclimative adjustments in Cd exposed roots appears to be long-lasting. The most conspicuous pattern apparent from the GO term enrichment analysis is the dominance of gene groups associated with plant defense. Several of the overrepresented GO terms among core up-regulated genes belong into the immunity context. The same is true when the analysis is restricted to the genes up-regulated both after 30 h^37^ and after 7 d (data reported here). Furthermore, this observation is in accordance with the finding that when different toxic ions (Al^3+^, Cu^2+^, Cd^2+^, Na^+^) were analyzed for their effect on the *A. thaliana* root transcriptome, defense genes were enriched in the group of genes specifically responsive to Cd stress^40^. The functional significance of this congruence in transcriptome changes remains unclear. Possibly the type of oxidative stress caused by Cd exposure^41^ bears similarities with the elevated ROS production typical for immune responses. Alternatively, it might be more than an oddity that there are several proteins implicated in both metal tolerance and plant nonhost resistance, e.g. ABCG36/PDR8/PEN3^42, 43^ and AtPCS1^44^.

Apparently not sustained over longer exposure times is one of the major acclimations of plants to Cd stress, namely the up-regulation of sulfur assimilation. There is ample evidence from transcriptome, proteome and metabolome studies that in order to replenish glutathione consumed for phytochelatin synthesis^45^, sulfate uptake and activation, the reduction of adenosine phosphosulfate as well as the synthesis of cysteine are strongly and rapidly activated^39, 40, 46, 47^. However, similar to the findings of van de Mortel *et al*.^48^ for *A. thaliana* roots exposed to Cd for one week as well, sulfur assimilation genes are not enriched among the responsive genes. While single members of the SULTR sulfate transporter family (e.g. SULTR2;1) are still up-regulated after 7 d, the enrichment of the GO term “sulfur compound metabolic process” (Fig. 7) is attributable rather to genes encoding glutathione S-transferases. Another sulfur metabolism-related long-term change is the down-regulation of glucosinolate biosynthesis. This has been hypothesized as a mechanism to make more sulfur available for PC synthesis^37^ and according to our data indeed represents a sustained core response of Cd-exposed *A. thaliana* roots.

The analysis of accession-specific responses suggested a second possible explanation for the higher Cd tolerance of Bur-0 besides the reduced Cd accumulation (Fig. 4a). Bur-0 roots responded more strongly to Cd than Col-0 even though according to the PC accumulation data there was no difference in the degree of effective Cd exposure, i.e. available Cd in the cytosol. The stronger response is not only apparent in the larger number of up- and down-regulated genes. Many of the core response GO terms as well as several other related GO terms, mainly additional defense-associated biological processes such as “response to chitin”, “innate immune response” and “defense response to bacterium”, were enriched among the genes responsive to Cd only in Bur-0. Thus, a possible explanation for the higher Cd tolerance of Bur-0 could be a more efficient activation of acclimative transcriptome changes. The understanding of signal transduction events mediating Cd responses is still fragmentary. Recently, the transcription factor ZAT6 was assigned a role in the activation of genes involved in the phytochelatin pathway^49^. Overexpression of ZAT6 conferred higher Cd tolerance. Interestingly, *ZAT6* and one of its target genes (*ABCG36*/*PDR8*/*PEN3*) as well as many other genes associated with Cd tolerance, e.g. SULTR3;4, SULTR4;1, SBP1^50^, ABCC2^51^, and ABCC3^52^ were up-regulated specifically in Bur-0. Thus, there could be a difference between Bur-0 and Col-0 in the events upstream of ZAT6 activation. Responsible genes in the mapped Cd tolerance QTLs will hopefully be identified when additional information becomes available on gene functions and on chromosomal regions influencing variation in Cd tolerance across other *A. thaliana* populations.

## Methods

### *Arabidopsis thaliana* lines

*A. thaliana* accessions were obtained from the Nottingham Arabidopsis Stock Center (NASC). The Col-0 × Bur-0 RIL population was obtained from the Versailles Arabidopsis Stock Center and was developed by Simon *et al*.^22^. The core population of 164 RILs was used for the mapping-related phenotyping.

### Cultivation conditions

Plants were cultivated in 1/10 Hoagland medium under long day conditions of 16 h light (50 µE) /8 h dark in a cultivation room. For the assessment of metal tolerance phenotypes a medium solidified with 1% (w/v) agarose (Type A, Sigma Aldrich) was used. The nutrient solution contained 100 µM (NH_4_)_2_HPO_4_, 200 µM MgSO_4_, 280 µM Ca(NO_3_)_2_, 600 µM KNO_3_, 5 µM Fe-HBED and 1% (w/v) sucrose and was buffered with 5 mM MES at pH 5.7. Metal salts were added as indicated.

For hydroponic culture plants were grown in a modified, liquid 1/10 Hoagland medium (87.1 µM (NH_4_)_2_HPO_4_, 200 µM MgSO_4_, 400 µM Ca(NO_3_)_2_, 600 µM KNO_3_, 5 µM Fe-HBED, 4.63 µM H_3_BO_3_, 0.032 µM CuSO_4_, 0.915 µM MnCl_2_, 0.077 µM ZnSO_4_, 0.011 µM MoO_3_; pH 5.7). Starting at week 2 of the plant cultivation the Bur-0 and Col-0 individuals were kept in 50 ml Falcon tubes. During the 3^rd^ and 4^th^ week the medium was exchanged once per week. From week 5 on the medium was exchanged twice per week. The plants were cultivated in climate chambers at short day conditions of 8 h light (200 µE)/16 h dark with 22 °C during the light period and 20 °C during the dark period.

### Soil experiments

Experiments were performed as described previously^53^. Control soil was spiked with 400 mg ZnSO_4_ per kg soil (dry weight) and Zn contaminated soil was prepared by adding 6400 mg ZnSO_4_ per kg soil.

### Phenotyping

In order to determine the Cd tolerance of hydroponically grown plants, the leaf growth was monitored by determining leaf area over the course of the cultivation. For the quantification of the leaf area, pictures were taken (same distance from the objects and a marker of known size) and analyzed (Adobe Photoshop CS2, version 9.0). Pixels of similar color were selected by use of the “Magic Wand Tool” at a tolerance of 32 using the neighboring and smoothing tool. Pixels of the leaf area were normalized to the pixels covering a standard of known size.

### QTL mapping

164 RILs were phenotyped under control conditions and in the presence of 2 µM CdCl_2_. The relative root growth (RRG) was calculated for each RIL as the mean root length on Cd plates normalized to the mean root length on control plates. RRG and genotypic information^22^ were loaded into the program MapQTL5®^54^. Standard methods for interval mapping^55^ were applied and the LOD score for each position on the genome was determined as an output for the likelihood of the association of a particular genomic region with the Cd tolerance phenotype.

### PCR based marker analysis

For further genotyping of interesting RILs, gDNA was extracted from young leaves or seedlings. To 100 mg pulverized, frozen plant material 500 µl extraction buffer (200 mM Tris/HCl pH 7.5, 250 mM NaCl, 25 mM EDTA, 0.5% SDS) were added. The DNA contained in the aqueous phase was then precipitated using isopropanol (1:1 ratio of DNA fraction to ethanol) and washed with ethanol. After resuspension in 50 µl deionized water, gDNA was used for PCR amplification. PCR was performed using the following protocol: 94 °C 1 min initial denaturation followed by 35 cycles of 94 °C 30 sec; X °C 30 sec; 72 °C 1 min/1 kb followed by a final elongation step at 72 °C for 3 min. The annealing temperature X was chosen for each primer pair 2 °C below the lower T_m_ of the two primers. Four CAPS markers were generated between the markers *2900 and *4011 (*3109 fw: TTTCCTTACAAGCCTGAAATATCC, rev: AAACACATCGCACATTTTGAAC, flanking a *Bse*LI restriction site specific for Col-0; *3343 fw: GATGCCATAGAGATGAATGCAG, rev: ATGGGCCTATGTGTAATCAACC, flanking a *Taq*I restriction site specific for Bur-0; *3558 fw: TTCCTCAGATCATCCATGTGTC, rev: TGCAAATACTAATGCTCCAACG, flanking a *Kpn*I restriction site specific for Col-0; *3782 fw: TGTTGTGCCTTGATTTTCAGTC, rev: TGGGTGCAAATTTCTCCTCTAC, flanking a *Tas*I restriction site specific for Col-0) and 17 RILs were analyzed for their genotype in this region.

### Transcriptome analysis

Plants were grown hydroponically. After one week of Cd exposure, roots and shoots of 3-4 individuals were pooled and two pools for each treatment and accession were generated. Harvested plant material was immediately frozen in liquid nitrogen. Plants were harvested early in the morning at the beginning of the light period. RNA from root material was extracted by adding 1 ml of TRIzol® (Invitrogen) to 100 mg of frozen plant powder and, after mixing, adding 200 µl chloroform. From the resulting aqueous phase the RNA was precipitated using isopropanol in a 1:1 ratio. The washed RNA was resuspended in 20 µl RNase-free H_2_O. Further purification was achieved by using the RNaeasy Kit from Quiagen, following the instruction manual of the manufacturer.

For the microarray analysis the extracted RNA was translated into aRNA with the GeneChip® 3′ IVT Express Kit from Affymetrix. Fragmented aRNA was hybridized to an *Arabidopsis* GeneChip microarray^56^ at the „Zentum für medizinische Grundlagenforschung der Martin-Luther-Universität“ in Halle/Saale, Germany. An Affymetrix GeneChip Scanner 3000 was used to analyze the hybridized microarray, applying an Affymetrix GeneChip Operating Software (GCOS). Program R (version 3.0.0)^57^ and the Bioconductor packages^58^ were used for data normalization, pre-processing and quality control. Signals were normalized using the Robust Multi-chip Average (RMA) method at standard settings. A linear model was used to calculate fold changes and *P*_adj_. values for each gene. The lmFit function was used with standard settings. The affy package was employed for the analysis of the data, quality control and annotation. Differentially expressed genes were selected based on statistically significant (*P*_adj_ ≤ 0.05) expression differences of at least 2fold between the genotypes under equal conditions or between treatments within each genotype. GO enrichment was performed with the PANTHER classification system (database release 27.10.2016) developed by the Gene Ontology Consortium (Mi *et al*., 2013). Cluster analysis was performed using the software R (version 3.3.1). Raw data of the microarray experiment reported here can be accessed via the GEO (Gene Expression Omnibus) data base under the accession number GSE94314.

For quantitative real-time (qRT) analysis of *HMA3* transcript abundance cDNA was synthesized from DNaseI treated RNA extracted from seedlings, using the RevertAid First Strand cDNA Synthesis Kit (Thermo-Fisher). The qRT PCR was performed using SYBR Green (iQ SYBR Green supermix, Bio-Rad) in a 96-well iCycler with a MyiQ real-time RCR detection system. The master mix contained 5 pmol of forward and reverse primer (*HMA3*_Bur_fw: CCAAACTGCAATGCTCACAG; *HMA3*_Bur_rev: TGGCCCTTGGATCTTGAAATC) and the PCR product was amplified from 5 µl of a 1:50 dilution of cDNA in a total volume of 20 µl. The PCR program was 95 °C for 10 min, 40 cycles of 95 °C for 10 sec and 60 °C for 1 min. The relative transcript level was determined as 1000 × 2^−ΔCT^ with the cycle threshold difference ΔCT being CT_Target gene_ − CT_EF1α_.

### SIFT analysis

Insertions and deletions in the Bur-0 genome within the QTL interval on chromosome 5 were downloaded from the 1001 Genomes Data Center: (http://1001genomes.org/data/MPI/MPIOssowski2008/releases/current/strains/Bur-0/). Sequence differences between Col-0 and Bur-0 were validated using the 1001 genomes browser: http://signal.salk.edu/atg1001/3.0/gebrowser.php. The most recent information about single nucleotide polymorphisms (SNPs) between Col-0 and Bur-0, stemming from the resequencing efforts of the 1001 genomes project, were downloaded from the GWA-Portal: https://gwas.gmi.oeaw.ac.at/#/genotype/snpviewer. Line 7058 was chosen to extrapolate all SNP positions leading to amino acid differences in the protein sequences between Col-0 and Bur-0 within the QTL region on chromosome 5. Line 7058 was chosen since it was the line used in the resequencing efforts and has the most SNPs annotated. SNPs were used to predict potentially non-functional AA exchanges in Bur-0 using the SIFT4G application (Vaser *et al*., 2016).

### Phytochelatin analysis

Phytochelatins (PCs) were determined by UPLC-ESI-QTOF-MS as described earlier^53^. Briefly, frozen homogenized plant material was extracted with 0.1% (v/v) trifluoroacetic acid containing 6.3 mM diethylene triamine pentaacetic acid and 40.04 µM N-acetylcysteine as internal standard. Prior to derivatization with monobromobimane at 45 °C for 30 min, thiol groups were reduced with Tris-(2-carboxyethyl)-phosphine. Labelled thiols were separated on a HSS T3 column (1.8 μm, 2.1 × 100 mm; Waters Corporation, Milford, MA, USA) by a Waters Aquity UPLC system applying a linear binary gradient of water (A) and acetonitrile (B), both acidified with 0.1% (v/v) formic acid, at a flow of 0.5 mL min^–1^: 99.5% A, 0.5% B for 1 min, a linear gradient to 60.5% B at 10 min, gradient to 99.5% B at 12 min, flushing with 99.5% B for 1 min, a gradient back to initial conditions in 1 min and an additional re-equilibration for 1 min. The column temperature was set to 40 °C. Thiols were detected with a Q-TOF Premier mass spectrometer equipped with an ESI-source (Waters Corporation) operated in the V + mode. For quantification the QuanLynx module of the MarkerLynx software was used.

### Inductively Coupled Plasma-Optical Emission Spectroscopy

Dry plant material was, after washing at 4 °C with H_2_O (twice, 10 min), 100 mM CaCl_2_ (once, 10 min), 50 mM EDTA (once, 5 min), digested in 4 ml HNO_3_ and 2 ml H_2_O_2_ for 12 min at 180 °C using a microwave (Start 1500, MLS GmbH). Metal concentrations were measured via ICP-OES (ICAP 6500, Thermo): Zn at 213.8 nm, Fe at 238.2 nm, Cd at 226.5 nm and Mn at 257.6 nm.

## Electronic supplementary material

Supplementary Files

Supplementary dataset S1

Supplementary dataset S2
