# Supplementary material for: Natural variation in Arabidopsis thaliana Cd responses and the detection of quantitative trait loci affecting Cd tolerance
Source: Sci Rep. 2017 Jun 16;7:3693. doi: 10.1038/s41598-017-03540-z (PMC5473843; doi:10.1038/s41598-017-03540-z)
Supplement: Supplementary file 1 — Supplementary Files [file 41598_2017_3540_MOESM1_ESM.pdf]

Supplementary files

Natural variation in *Arabidopsis thaliana* Cd responses and the detection of quantitative trait loci affecting Cd tolerance

Sina Fischer[1,2], Thomas Spielau[1] and Stephan Clemens[1*]

[1]Department of Plant Physiology, and Bayreuth Center of Ecology and Environmental Research, University of Bayreuth, Universitätsstrasse 30, 95447 Bayreuth, Germany
[2]School of Biosciences, University of Nottingham, Sutton Bonington Campus, Loughborough, Leicestershire, LE12 5RD, UK

Supplementary Fig. S1: Natural variation in *A. thaliana* Cu tolerance.

Supplementary Fig. S2: Metal accumulation in Cd- or Zn-treated Col-0 and Bur-0 plants.

Supplementary Fig. S3: QTL mapping based on the relative seedling weight.

Supplementary Fig. S4: The $HMA3_{Bur\text{-}0}$ allele does not confer higher Cd tolerance to Col-0 or the Cd hypersensitive mutant *cad1-3*.

Supplementary Fig. S5: Physiological characterization of Col-0 and Bur-0 plants analyzed for root transcriptome changes upon Cd exposure.

Supplementary Fig. S6: Cluster analysis of differentially regulated genes of QTL5.

Supplementary Tab. S1: SIFT analysis of genes with nonsynonymous differences between Bur-0 and Col-0 in the QTL region on chromosome 5.

Supplementary Tab. S2: SIFT analysis of genes with nonsynonymous differences between Bur-0, Cvi-0 and St-0 versus Col-0 in the QTL region on chromosome 5

Supplementary Figure S1 (Fischer et al.)

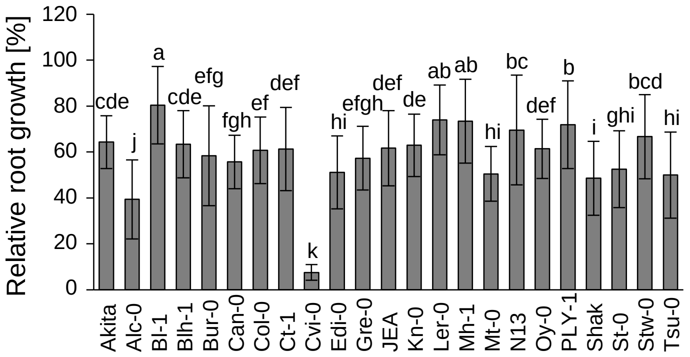

Supplementary Fig. S1: **Natural variation in *A. thaliana* Cu tolerance.** 23 accessions of the Versailles Core24 were grown on vertical agar plates under control conditions or in the presence of 10 µM $CuSO_4$. Length of primary roots was determined after 12-14 d. Relative root growth of seedlings as an indicator for tolerance was calculated as follows: root length metal treatment/mean root length control conditions x 100. Data represent means ±SD. Per accession 17 to 185 plants were compared. The average n was 119. Statistical analysis was performed via one-way ANOVA and data were grouped based on Tukey’s 95% confidence intervals.

Supplementary Figure S2 (Fischer et al.)

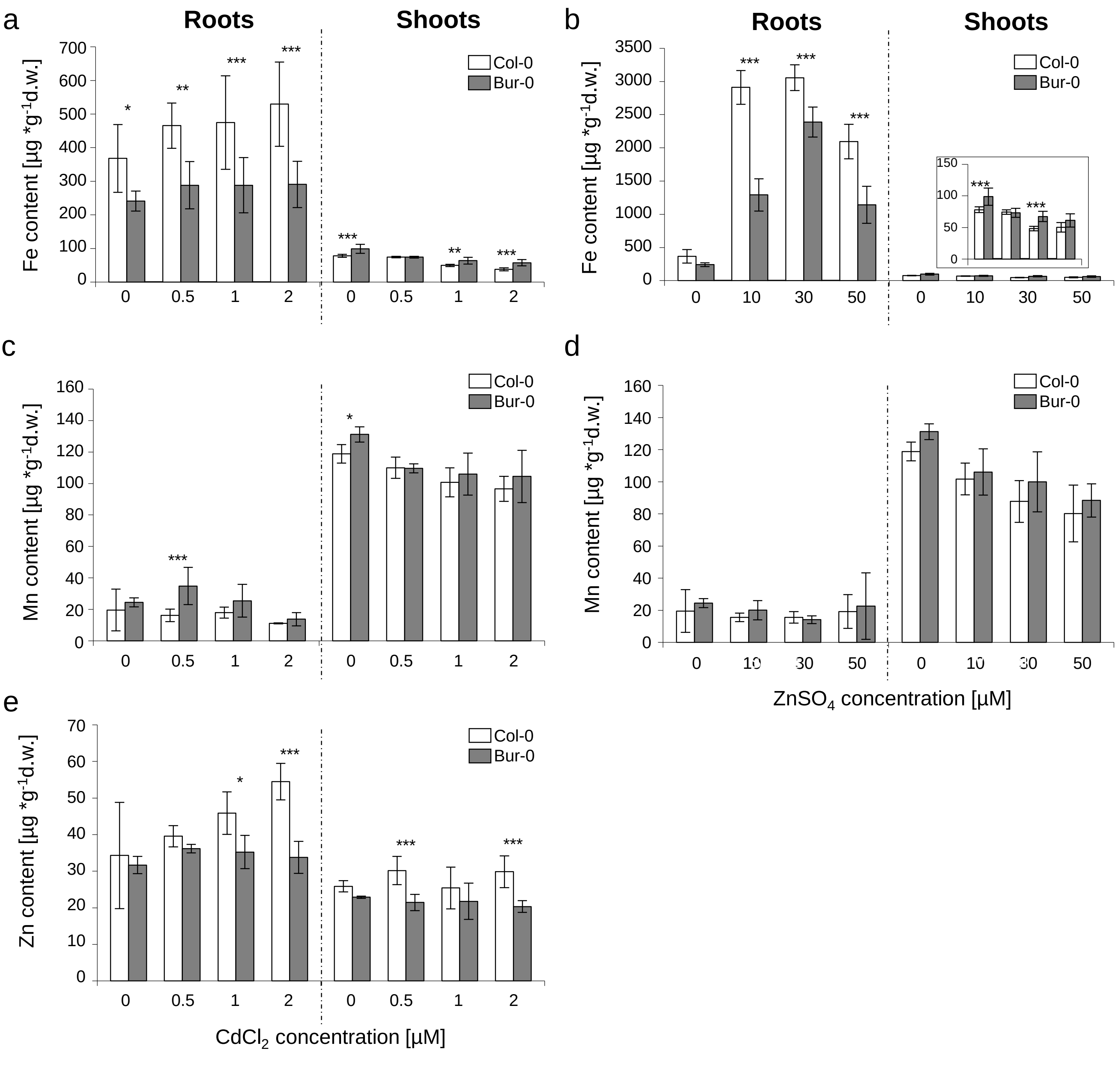

Supplementary Fig. S2: Metal accumulation in Cd- or Zn-treated Col-0 and Bur-0 plants. Fe (a, b), Mn (c, d) and Zn (e) content of hydroponically grown Col-0 (white bars) and Bur-0 (grey bars) plants was determined in roots and shoots after six weeks of growth. For the last week of cultivation, different concentrations of $CdCl_2$ (left) or $ZnSO_4$ (right) were added to the medium. Please note that Cd content of plants grown in the absence of $CdCl_2$ was below the detection limit. Data represent means ±SD of 3 independent experiments (n=6, i.e. two sample pools per plant line and condition). Statistical analysis was performed by one-way ANOVA and subsequent Tukey test. The asterisks indicate significant differences between the accessions (***, P<0.001; **, P<0.01; *, P<0.05), 95% confidence interval.

Supplementary Figure S3 (Fischer et al.)

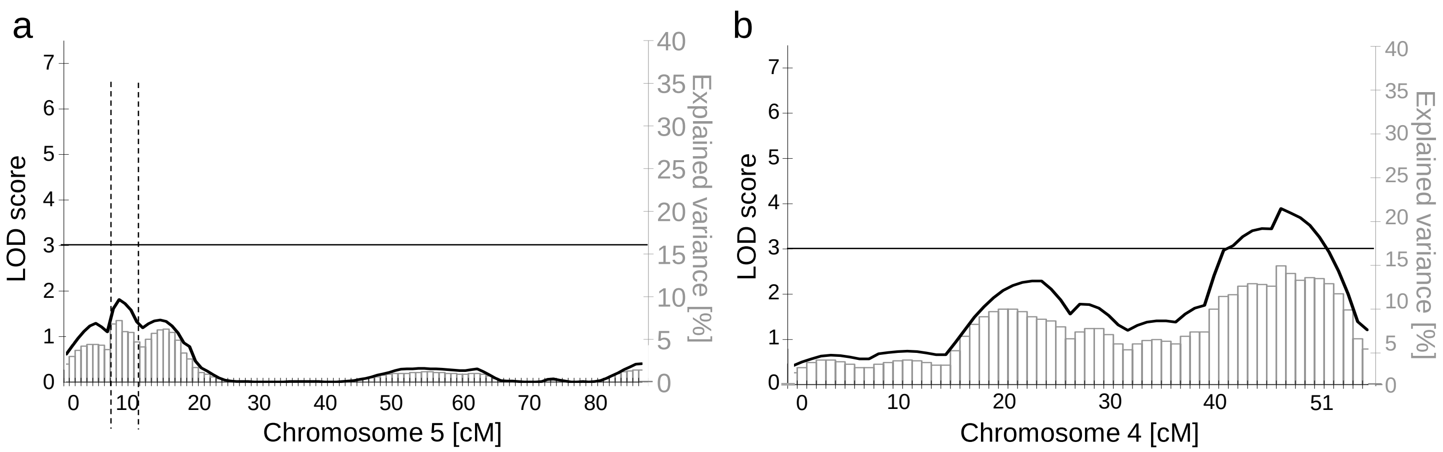

Supplementary Fig. S3: **QTL mapping based on the relative seedling weight.** After cultivation of 131 RILs at 2 µM $CdCl_2$ QTLs were detected on chromosome 5 (a) and on chromosome 4 (b). Dotted vertical lies show the position of the highest LOD score for the RRG (see Fig. 6) to be overlapping with the region quantified by relative seedling weight. The horizontal line indicates the threshold of LOD 3, used to indicate the 95% confidence interval.

Supplementary Figure S4 (Fischer et al.)

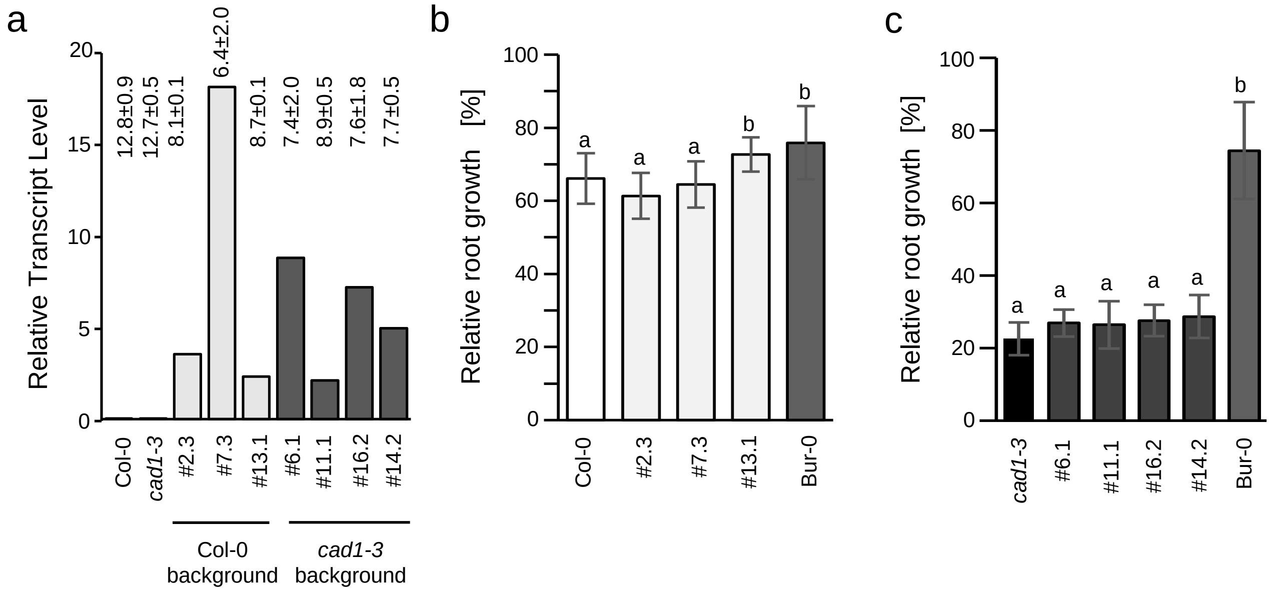

Supplementary Fig. S4: The *HMA3*$_{\text{Bur-0}}$ allele does not confer higher Cd tolerance to Col-0 or the Cd hypersensitive mutant *cad1-3*. Three independent transgenic Col-0 lines and 4 independent transgenic *cad1-3* lines transformed with a genomic version of the Bur-0 *HMA3* allele were randomly chosen and analyzed for *HMA3* expression. (a) The mean relative transcript level (RTL = $2^{-\Delta CT}$x1000) is shown with mean ΔCT± standard deviation of two independent experiments. The Cd tolerance was assessed for the lines in Col-0 background (b) and *cad1-3* background (c) in comparison with Bur-0 and the respective untransformed lines by growing seedlings under control conditions and in the presence of 2 µM $CdCl_2$ or 1 µM $CdCl_2$,respectively. Relative root growth of seedlings as an indicator for tolerance was calculated as follows: root length metal treatment/mean root length control conditions x 100. Data were derived from 2 independent experiments with n=14-75 plants per genotype and condition. Statistical analysis was performed via one-way ANOVA and data were grouped based on Tukey's 95% confidence intervals.

Supplementary Figure S5 (Fischer et al.)

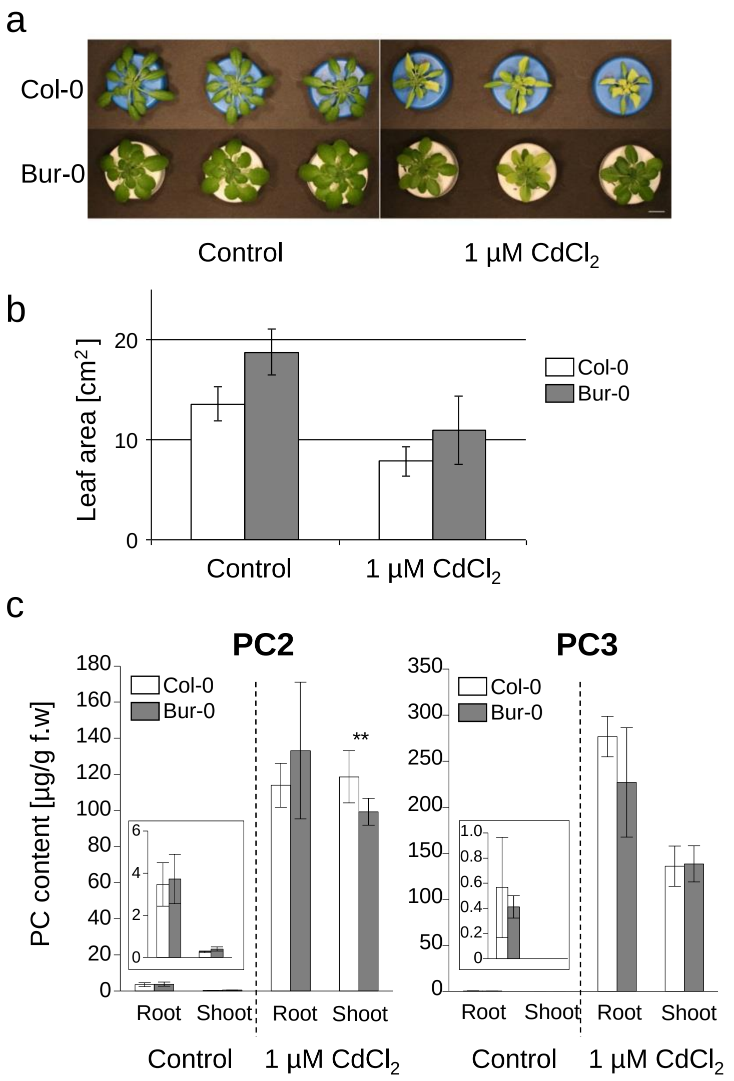

Supplementary Fig. S5: **Physiological characterization of Col-0 and Bur-0 plants analyzed for root transcriptome changes upon Cd exposure.** Hydroponically grown Bur and Col plants were treated with 1 µM $CdCl_2$ for one week. This resulted in comparable growth reduction as indicated by leaf area (a, b). (c) Cd-exposed roots showed a similar degree of Cd-activated phytochelatin accumulation (PC2 and PC3). Data represent means ±SD of 4 independent experiments. Statistical analysis was performed by one-way ANOVA and subsequent Tukey test. The asterisks indicate significant differences between the accessions (**, $P<0.01$), 95% confidence interval.

Supplementary Figure S6 (Fischer et al.)

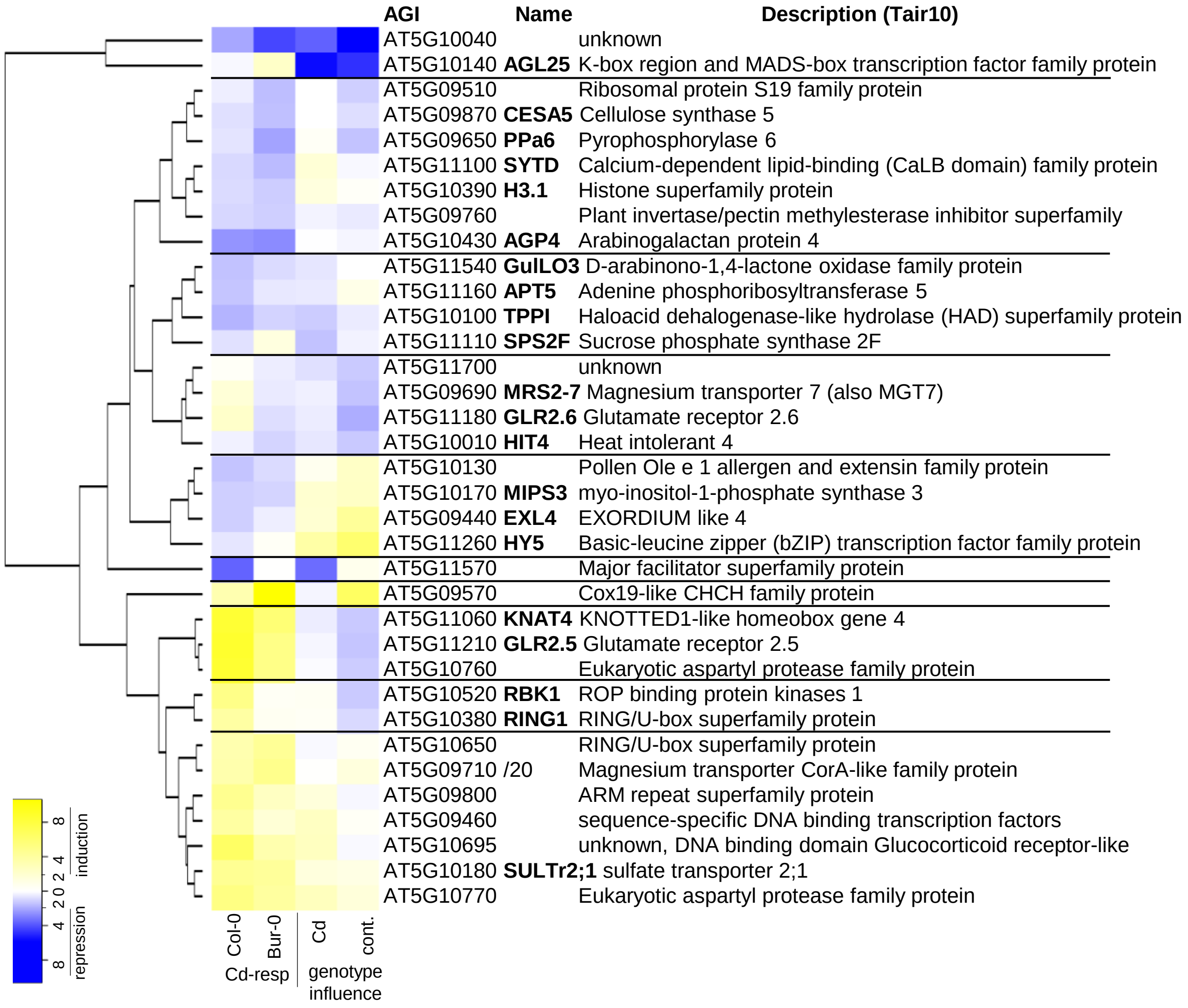

Supplementary Fig. S6: **Cluster analysis of differentially regulated genes of QTL5.** Cluster analysis (performed with R version 3.3.1) of all genes within the QTL region on chromosome 5 from 2.9 to 3.84 Mb that showed a significant 2fold expression difference between treatments or genotypes. Scale indicates fold changes, yellow at least 2fold upregulation upon Cd-treatment or in Bur-0 compared to Col-0, blue at least 2fold downregulation upon Cd-treatment or in Bur-0 compared to Col-0.

Supplementary Table S1 (Fischer et al.)

Supplementary Table S1: SIFT analysis of genes with nonsynonymous differences between Bur-0 and Col-0 in the QTL region on chromosome 5.

AGI	Description	Alias	accession	Position on Chr. 5	effect	SIFT prediction (*WARNING! low confidence)
functional in Bur-0 but not in Col-0						
AT5G09690	magnesium transporter 7	MGT7	Bur-0	3002302	AA exchange: F to S	TOLERATED
			Col-0	3002302	original AA: F	DELETERIOUS
AT5G11250	Disease resistance protein (TIR-NBS-LRR class)		Bur-0	3591386	AA exchange: W to L	TOLERATED
			Col-0	3591386	original AA: W	DELETERIOUS
functional in Col-0 but not in Bur-0						
AT5G09410	ethylene induced calmodulin binding protein	EICBP.B	Bur-0	2921436	AA exchange: D to N	DELETERIOUS *
			Bur-0	2926859	AA exchange: L to F	DELETERIOUS
AT5G09420	translocon at the outer membrane of chloroplasts 64-V	TOC64-V	Bur-0	2928544	AA exchange: S to T	DELETERIOUS
AT5G09450	Tetratricopeptide repeat (TPR)-like superfamily protein		Bur-0	2942542	AA exchange: D to H	DELETERIOUS
AT5G09580	heat shock protein		Bur-0	2972814	AA exchange: A to G	DELETERIOUS
AT5G09610	pumilio 21	PUM21	Bur-0	2981652	AA exchange: S to F	DELETERIOUS *
AT5G09680	reduced lateral root formation	RLF	Bur-0	2999387	AA exchange: T to I	DELETERIOUS *
AT5G09700	Glycosyl hydrolase family protein		Bur-0	3003953	AA exchange: E to Q	DELETERIOUS
			Bur-0	3004198	AA exchange: Y to F	DELETERIOUS
			Bur-0	3004551	AA exchange: P to Q	DELETERIOUS
AT5G09720	Magnesium transporter CorA-like family protein		Bur-0	3012631	1 base deletion in exon	
			Bur-0	3013430	AA exchange: L to F	DELETERIOUS
			Bur-0	3014186	AA exchange: V to I	DELETERIOUS
			Bur-0	3014216	AA exchange: S to P	DELETERIOUS
AT5G09850	Transcription elongation factor (TFIIS) family protein		Bur-0	3064235	AA exchange: S to P	DELETERIOUS
AT5G09870	cellulose synthase 5	CESA5	Bur-0	3074802	AA exchange: R to M	DELETERIOUS
AT5G09978	elicitor peptide 7 precursor	PEP7	Bur-0	3120756	AA exchange: G to E	DELETERIOUS
AT5G09995	transmembrane protein		Bur-0	3126065	AA exchange: G to R	DELETERIOUS
AT5G10040	transmembrane protein		Bur-0	3143919	1 base insertion in Exon	
AT5G10060	ENTH/VHS family protein		Bur-0	3147133	AA exchange: G to V	DELETERIOUS
AT5G10070	RNase L inhibitor protein-like protein		Bur-0	3148796	AA exchange: D to A	DELETERIOUS
AT5G10120	Ethylene insensitive 3 family protein		Bur-0	3170090	AA exchange: Q to P	DELETERIOUS
AT5G10210	nitric oxide synthase-interacting protein		Bur-0	3206360	AA exchange: N to S	DELETERIOUS
			Bur-0	3206627	AA exchange: V to E	DELETERIOUS
AT5G10220	annexin 6	ANN6	Bur-0	3207655	AA exchange: Y to N	DELETERIOUS
AT5G10230	annexin 7	ANNAT7	Bur-0	3210138	2 base insertion in Exon	
AT5G10250	Phototropic-responsive NPH3 family protein	DOT3	Bur-0	3218439	AA exchange: R to H	DELETERIOUS
AT5G10440	cyclin d4;2	CYCD4;2	Bur-0	3281712	AA exchange: Q to H	DELETERIOUS
AT5G10590	hypothetical protein		Bur-0	3349145	1 base insertion in Exon	
			Bur-0	3349163	AA exchange: I to L	DELETERIOUS *
AT5G10630	Translation elongation factor EF1A/initiation factor IF2gamma family protein		Bur-0	3360634	AA exchange: Y to C	DELETERIOUS
			Bur-0	3361543	AA exchange: N to Y	DELETERIOUS
			Bur-0	3362055	AA exchange: D to N	DELETERIOUS
			Bur-0	3362260	AA exchange: K to T	DELETERIOUS
AT5G10660	calmodulin-binding protein-like protein		Bur-0	3372242	AA exchange: K to T	DELETERIOUS *
AT5G10690	pentatricopeptide (PPR) repeat-containing protein / CBS domain-containing protein		Bur-0	3377057	AA exchange: N to K	DELETERIOUS *
AT5G10770	Eukaryotic aspartyl protease family protein		Bur-0	3403657	AA exchange: A to T	DELETERIOUS
AT5G10800	RNA recognition motif (RRM)-containing protein		Bur-0	3412914	AA exchange: R to I	DELETERIOUS *
AT5G10880	tRNA synthetase-related / tRNA ligase-like protein		Bur-0	3433752	AA exchange: G to E	DELETERIOUS *
			Bur-0	3433832	AA exchange: A to P	DELETERIOUS *
AT5G11030	aberrant root formation protein	ALF4	Bur-0	3491931	AA exchange: E to K	DELETERIOUS
			Bur-0	3491965	AA exchange: C to W	DELETERIOUS
AT5G11080	Ubiquitin-like superfamily protein		Bur-0	3518690	AA exchange: T to I	DELETERIOUS *
AT5G11180	glutamate receptor 2.6	GLR2.6	Bur-0	3559864	AA exchange: E to G	DELETERIOUS
AT5G11210	glutamate receptor 2.5	GLR2.5	Bur-0	3573045	AA exchange: T to A	DELETERIOUS
AT5G11230	Nucleotide-sugar transporter family protein		Bur-0	3581292	AA exchange: C to S	DELETERIOUS
AT5G11350	DNAse I-like superfamily protein		Bur-0	3624489	AA exchange: T to I	DELETERIOUS
			Bur-0	3625188	AA exchange: R to C	DELETERIOUS
AT5G11400	Protein kinase superfamily protein		Bur-0	3637108	1 base deletion in exon	
AT5G11412	RNA-binding (RRM/RBD/RNP motifs) family protein		Bur-0	3640445	1 base insertion in Exon	
AT5G11416	hypothetical protein		Bur-0	3643009	AA exchange: M to R: Start lost!	DELETERIOUS *
AT5G11450	PsbP domain protein (Mog1/PsbP/DUF1795-like photosystem II reaction center PsbP family protein)	PPD5	Bur-0	3655222	AA exchange: R to S	DELETERIOUS
AT5G11460	hypothetical protein (DUF581)		Bur-0	3657562	AA exchange: Y to C	DELETERIOUS
AT5G11470	bromo-adjacent homology (BAH) domain-containing protein		Bur-0	3666881	AA exchange: P to L	DELETERIOUS *
AT5G11570	Major facilitator superfamily protein		Bur-0	3717340	AA exchange: L to V	DELETERIOUS
			Bur-0	3717904	AA exchange: I to T	DELETERIOUS
			Bur-0	3718231	AA exchange: G to C	DELETERIOUS
AT5G11580	Regulator of chromosome condensation (RCC1) family protein		Bur-0	3720137	AA exchange: D to A	DELETERIOUS
AT5G11610	Exostosin family protein		Bur-0	3737307	AA exchange: R to T	DELETERIOUS
AT5G11650	alpha/beta-Hydrolases superfamily protein		Bur-0	3746805	AA exchange: S to G	DELETERIOUS *
AT5G11670	NADP-malic enzyme 2	NADP-ME2	Bur-0	3755442	AA exchange: G to D	DELETERIOUS
AT5G11760	stress response protein		Bur-0	3789967	AA exchange: D to Y	DELETERIOUS
AT5G11850	Protein kinase superfamily protein		Bur-0	3820180	AA exchange: V to I	DELETERIOUS
AT5G11920	6-&1-fructan exohydrolase	cwINV6	Bur-0	3840297	AA exchange: P to L	DELETERIOUS
non-functional in Col-0 and in Bur-0						
AT5G09730	beta-xylosidase 3	BXL3	Bur-0	3016640	AA exchange: G to A	TOLERATED
			Col-0	3016640	original AA: G	DELETERIOUS
			Bur-0	3015801	AA exchange: S to C	DELETERIOUS
			Bur-0	3017348	AA exchange: N to H	DELETERIOUS

Supplementary Table S2 (Fischer et al.)

AGI	Description	Alias	accession	Position on Chr. 5	effect	SIFT prediction (*WARNING! low confidence)
	Supplementary Table S2: SIFT analysis of genes with nonsynonymous differences between Bur-0, Cvi-0 and St-0 versus Col-0 in the QTL region on chromosome 5					
functional in Bur-0, Cvi-0 and St-0 but not in Col-0						
AT5G09690	magnesium transporter 7	MGT7	Cvi-0	3002303	AA exchange: F to S	TOLERATED
			St-0	3002304	AA exchange: F to S	TOLERATED
			Col-0	3002302	original AA: F	DELETERIOUS
functional in Cvi-0 or St-0 but not in Col-0						
AT5G09730	beta-xylosidase 3	BXL3	Cvi-0	3016640	AA exchange: G to A	TOLERATED
			Col-0	3016640	original AA: F	DELETERIOUS
AT5G10420	MATE efflux family protein		Cvi-0	3276056	AA exchange: E to K	TOLERATED
			Col-0	3276056	original AA: F	DELETERIOUS
AT5G10560	Glycosyl hydrolase family protein	BXL6	Cvi-0	3339170	AA exchange: M to T	TOLERATED
			St-0	3339170	AA exchange: M to T	TOLERATED
			Col-0	3339170	original AA: F	DELETERIOUS
AT5G11250	Disease resistance protein (TIR-NBS-LRR class)		Cvi-0	3591386	AA exchange: W to L	TOLERATED
			St-0	3591386	AA exchange: W to L	TOLERATED
			Col-0	3591386	original AA: F	DELETERIOUS
functional in Col-0 but not in Bur-0 and Cvi-0 and St-0						
AT5G09450	Tetratricopeptide repeat (TPR)-like superfamily protein		Cvi-0	2942542	nonsynonymous AA exchange from D to H	DELETERIOUS
			St-0	2942542	nonsynonymous AA exchange from D to H	DELETERIOUS
AT5G09700	Glycosyl hydrolase family protein		Cvi-0	3003953	nonsynonymous AA exchange from E to Q	DELETERIOUS
			St-0	3003953	nonsynonymous AA exchange from E to Q	DELETERIOUS
			St-0	3003992	STOP-GAIN, AA exchange from Q to *	
			Cvi-0	3004198	nonsynonymous AA exchange from Y to F	DELETERIOUS
			St-0	3004198	nonsynonymous AA exchange from Y to F	DELETERIOUS
			Cvi-0	3004551	nonsynonymous AA exchange from P to Q	DELETERIOUS
			St-0	3004551	nonsynonymous AA exchange from P to Q	DELETERIOUS
			Cvi-0	3004624	nonsynonymous AA exchange from D to Y	DELETERIOUS
			St-0	3004624	nonsynonymous AA exchange from D to Y	DELETERIOUS
			St-0	3004940	nonsynonymous AA exchange from I to M	DELETERIOUS
			Cvi-0	3005394	nonsynonymous AA exchange from T to M	DELETERIOUS
			St-0	3005394	nonsynonymous AA exchange from T to M	DELETERIOUS
AT5G09978	elicitor peptide 7 precursor	PEP7	Cvi-0	3120756	nonsynonymous AA exchange from G to E	DELETERIOUS
			St-0	3120756	nonsynonymous AA exchange from G to E	DELETERIOUS
AT5G10250	Phototropic-responsive NPH3 family protein	DOT3	Cvi-0	3217804	nonsynonymous AA exchange from I to T	DELETERIOUS
			Cvi-0	3218398	nonsynonymous AA exchange from A to S	DELETERIOUS
			St-0	3218617	nonsynonymous AA exchange from W to R	DELETERIOUS
AT5G10590			Cvi-0	3349389	nonsynonymous AA exchange from I to S	DELETERIOUS *
			St-0	3349389	nonsynonymous AA exchange from I to S	DELETERIOUS *
			Cvi-0	3349427	nonsynonymous AA exchange from K to N	DELETERIOUS *
			St-0	3349427	nonsynonymous AA exchange from K to N	DELETERIOUS *
			Cvi-0	3349456	nonsynonymous AA exchange from S to T	DELETERIOUS *
			St-0	3349456	nonsynonymous AA exchange from S to T	DELETERIOUS *
AT5G10800	RNA recognition motif (RRM)-containing protein		Cvi-0	3412914	nonsynonymous AA exchange from R to I	DELETERIOUS *
			St-0	3413152	nonsynonymous AA exchange from S to P	DELETERIOUS
			St-0	3413186	nonsynonymous AA exchange from R to S	DELETERIOUS
			St-0	3414696	nonsynonymous AA exchange from S to I	DELETERIOUS
			St-0	3414706	nonsynonymous AA exchange from I to F	DELETERIOUS
			St-0	3417311	STOP-GAIN, AA exchange from R to *	
			St-0	3433791	nonsynonymous AA exchange from E to G	DELETERIOUS *
AT5G11180	glutamate receptor 2.6	ATGLR2.6	St-0	3557799	nonsynonymous AA exchange from L to H	DELETERIOUS
		ATGLR2.6	Cvi-0	3559864	nonsynonymous AA exchange from E to G	DELETERIOUS
		ATGLR2.6	St-0	3560096	nonsynonymous AA exchange from E to K	DELETERIOUS
		ATGLR2.6	St-0	3560257	STOP-GAIN, AA exchange from L to *	
AT5G11210	glutamate receptor 2.5	ATGLR2.5	Cvi-0	3573045	nonsynonymous AA exchange from T to A	DELETERIOUS
		ATGLR2.5	St-0	3573045	nonsynonymous AA exchange from T to A	DELETERIOUS
		ATGLR2.5	Cvi-0	3573695	nonsynonymous AA exchange from G to D	DELETERIOUS
AT5G11416			Cvi-0	3643009	START-LOST, AA exchange from M to R	DELETERIOUS *
			St-0	3643009	START-LOST, AA exchange from M to R	DELETERIOUS *
AT5G11470	bromo-adjacent homology (BAH) domain-containing protein		Cvi-0	3663687	nonsynonymous AA exchange from G to E	DELETERIOUS *
			St-0	3663687	nonsynonymous AA exchange from G to E	DELETERIOUS *
			Cvi-0	3666881	nonsynonymous AA exchange from P to L	DELETERIOUS *
AT5G11650	alpha/beta-Hydrolases superfamily protein		Cvi-0	3746805	nonsynonymous AA exchange from S to G	DELETERIOUS *
			St-0	3746805	nonsynonymous AA exchange from S to G	DELETERIOUS *
